# Multi/Many-Objective Particle Swarm Optimization Algorithm Based on Competition Mechanism

**DOI:** 10.1155/2020/5132803

**Published:** 2020-02-19

**Authors:** Wusi Yang, Li Chen, Yi Wang, Maosheng Zhang

**Affiliations:** ^1^School of Information Technology and Software, Northwest University, Xi'an 710127, China; ^2^Key Laboratory for Geo-Hazards in Loess Area, MNR, Xi'an Center of Geological Survey, China Geological Survey, Xi'an 710054, China

## Abstract

The recently proposed multiobjective particle swarm optimization algorithm based on competition mechanism algorithm cannot effectively deal with many-objective optimization problems, which is characterized by relatively poor convergence and diversity, and long computing runtime. In this paper, a novel multi/many-objective particle swarm optimization algorithm based on competition mechanism is proposed, which maintains population diversity by the maximum and minimum angle between ordinary and extreme individuals. And the recently proposed *θ*-dominance is adopted to further enhance the performance of the algorithm. The proposed algorithm is evaluated on the standard benchmark problems DTLZ, WFG, and UF1-9 and compared with the four recently proposed multiobjective particle swarm optimization algorithms and four state-of-the-art many-objective evolutionary optimization algorithms. The experimental results indicate that the proposed algorithm has better convergence and diversity, and its performance is superior to other comparative algorithms on most test instances.

## 1. Introduction

Multiobjective optimization problem is generated from engineering application [1], controller optimization [2], economic scheduling [3], etc. When the number of objective is more than 3, it is defined as many-objective optimization problem (MaOP). The two main goals of solving multi/many-objective optimization problems are convergence and diversity of solutions. The purpose of convergence is to find a set of solutions as close as possible to the true Pareto front (PF) and diversity is to find a set of solutions as diverse as possible. Convergence to the true PF and maintaining a good diversity between solutions are two major difficulties in solving problems of multi/many-objective optimization. However, when solving the many-objective optimization problem, the number of multiple objectives needs to be optimized simultaneously and both the objective search space and the Pareto nondominance solutions increase exponentially. Moreover, the complexity of nondominated sorting increases dramatically. It is easy to find that the following problems commonly exist in solving many-objective optimization problems, such as poor performance, high-computational complexity, obtained approximate solution cannot approximate the true PF, uneven distribution, incomplete coverage, and poor stability. In recent years, many scholars have proposed many effective methods to solve many-objective optimization problems and improve the performance of the algorithm. The research of many-objective optimization algorithms can be summarized as follows.

The decomposition strategy-based algorithm mainly uses the aggregation method to transform the multi/many-objective problem into a single-objective optimization problem, such as MOEA/D [4], MOEA/D-AED [5], and MPSOD [6]. In the performance indicator-based algorithm, performance indicators of solution quality measurement are adopted as selection criteria in the environmental selection, such as HypE [7], MOMBI-II [8], and MaOEA-IGD [9]. The reference point guidance-based algorithm uses a set of reference points to guide evolution, evaluate the quality of the solution, and control the distribution of the population in the objective space, such as NSGA-III [10] and RVEA [11]. The many-objective optimization algorithm based on reduction of the objective number improves the optimization ability of the algorithm by reducing the objective dimension [12]. The loose Pareto dominance-based algorithm enhances the selection pressure of the algorithm by relaxing the dominance relationship in the selection of environment and strengthening the dominance relationship among individuals. Representatives of this type include *α*-dominance [13], fuzzy-dominance [14], *r*-dominance [15], and *ε*-dominance [5, 16].

In recent years, with the development of metaheuristic optimization algorithms based on different swarms, a large number of multiobjective optimization algorithms have been developed, such as firefly algorithm [17], bat searching algorithm [18], chicken swarm optimization algorithm [19], and artificial bee colony algorithm [20]. Particle swarm optimization (PSO) is an important metaheuristic optimization technology, which has been used to tackle multiobjective optimization problems and shows promising performance [21–23]. However, the multiobjective optimization algorithm based on particle swarm optimization is easy to fall into the local optimum. It is very important for the performance of multiobjective particle swarm optimization to achieve a reasonable balance between convergence and diversity. Generally, the optimal individual search of multiobjective particle swarm optimization is based on the assumption that no particle can achieve the best results on all the objectives of the multiobjective optimization problem. For this premise, there are several ways to update the speed and position of the PSO. In addition to the traditional methods of updating speed and location, there are other efficient methods. The double search strategy [24] is adopted to improve the velocity of updating particles, which are aimed at accelerating the convergence speed and maintaining the population diversity, respectively. As a variant of PSO, the competitive swarm optimization operator [25] is different from the traditional updating method mainly in which the competitors in the current group lead the search process rather than the historical location. And the competitive swarm optimization algorithm can achieve better results between convergence and diversity too. In addition, comprehensive learning particle swarm optimization uses the best historical information of particles to update the speed of particles, and this strategy can keep the diversity of the swarm and prevent premature convergence. The multiswarm comprehensive learning particle swarm optimization (MSCLPSO [26]) algorithm uses this strategy and combines multiple swarms to update the position of the particles, achieving better results.

Many papers have proposed different multiobjective particle swarm optimization algorithms, but most algorithms perform poorly while tackling MaOP. It is mainly due to the insufficient selection pressure of the algorithm, which cannot effectively balance the relationship between convergence and diversity, and makes the algorithm fall into the local area, far away from the true Pareto front. For example, the CMOPSO algorithm proposed in [27], which updates the population based on competitive-mechanism learning, can effectively tackle multiobjective optimization problems. However, experiments show that CMOPSO performs poorly in tackling many-objective optimization problems, and the algorithm generally runs for a long time on test problems. For the shortcomings of CMOPSO algorithm, we adopted a new environment selection strategy to maintain the diversity of population and employed *θ*-dominance sorting [28] and reference points regeneration strategy [11] to further enhance the performance of algorithm. A multi/many-objective particle swarm optimization algorithm based on competition mechanism is proposed, which is denoted as CMaPSO. The main contribution of this paper is summarized as follows:

1. In this paper, the recently proposed competitive mechanism-based learning is adopted to deal with many-objective optimization problems for the first time.
2. A new environment selection strategy to maintain the convergence and diversity of the algorithm and the optimal individuals are selected by using the maximum and minimum angle between the ordinary individuals and the extreme points.
3. The proposed algorithm CMaPSO are compared with four recently published multi/many-objective particle swarm optimization algorithms MMOPSO [24], MPSOD [6], CMOPSO [27], and dMOPSO [29]. Moreover, CMaPSO algorithm is compared with four state-of-the-art many-objective optimization algorithms, MOMBIII [8], NSGA-III [10], RPEA [30], and MyODEMR [31] on the standard benchmark problems to evaluate the performance of algorithms. Experimental results show that the performance of the proposed algorithm is better than the comparative algorithms on most standard benchmark problems.

The remainder of this paper is organized as follows.  Section 2 introduces the related background. The proposed CMaPSO is described in detail in Section 3.  Section 4 presents the benchmark MaOPs, performance metrics, and algorithm settings used for performance comparison. Finally, conclusion and future work are presented in Section 5.

## 2. Related Work

In this section, we introduce some concepts, including the definition of multiobjective optimization and particle swarm optimization. And we give a brief overview of the recently proposed algorithm *θ*-DEA [28]. Finally, several recently proposed multi/many-objective particle swarm optimization algorithms are summarized.

### 2.1. Multiobjective Optimization Problems

A general multiobjective optimization minimization problems can be formulated as follows [32]:

(1) $$\begin{array}{c} \begin{array}{cc} \text{minimize} & F\left(x\right)=\left(f_{1}\left(x\right),f_{2}\left(x\right),\ldots ,f_{m}\left(x\right)\right)\\ \text{subject to} & \begin{array}{c} g_{i}\left(x\right)\leq 0,i=1,2,\ldots ,p\\ h_{i}\left(x\right)=0,i=1,2,\ldots ,q, \end{array} \end{array} \end{array}$$

where *x*=(*x*_1_, *x*_2_,…, *x*_*n*_) ∈ *X* ⊂ *R*^*n*^ is an *n*-dimensional decision vector bounded in the decision space *X*, *F*(*x*) represents the *m*-dimensional objective vector, *f*_*i*_(*x*) represents the *i*th minimized objective function, and *g*_*i*_(*x*) and *h*_*i*_(*x*) are the constraint function of the problem. Several definitions related to multiobjective optimization are shown as follows.

**Definition 1 .**

(Pareto dominance). A decision vector *x* is said to dominate another decision vector *y*, denoted by *x*≺*y*, if and only if

(2) $$\begin{array}{c} \forall i\in \left\{1,2,\ldots ,m\right\}:f_{i}\left(x\right)\leq f_{j}\left(y\right)\wedge ,\\ \exists j\in \left\{1,2,\ldots ,m\right\}:f_{i}\left(x\right)<f_{j}\left(y\right). \end{array}$$

**Definition 2 .**

(Pareto optimal). A vector *x*^⋇^ ∈ *X* is Pareto optimal if there is no other *x* ∈ *X*, such that *x*≺*x*^⋇^.

**Definition 3 .**

(Pareto optimal set). For a given multiobjective optimization problem, the Pareto set is defined by the following equation:

(3) $$\begin{array}{c} PS=\left\{\left.x\in X\right|∄z\in X,z≺x\right\}. \end{array}$$

**Definition 4 .**

(Pareto front). For a given multiobjective optimization problem, the Pareto front is defined as follows:

(4) $$\begin{array}{c} PS=\left\{\left.F\left(X\right)\right|x\in PS\right\}. \end{array}$$

### 2.2. Particle Swarm Optimization

A stochastic algorithm based on bionic swarm intelligence named particle swarm optimization (PSO) [33] is proposed by Kennedy and Eberhart, which simulates the predation behavior of birds and fish. It seeks the optimal solution through the cooperation and information sharing among individuals in the swarm. At present, it has been widely used in function optimization, neural network training, fuzzy system control, and other application fields. Each particle represents a potential solution for the optimization problem, which is influenced by position and moving velocity. The velocity and position of the *k*th particle are represented as *v*_*k*_=(*v*_*k*1_,…, *v*_*km*_) and *x*_*k*_=(*x*_*k*1_,…, *x*_*km*_), respectively. The velocity and position of the *k*th particle are updated by the following function:

(5) $$\begin{array}{c} v_{kj}\left(t+1\right)=wv_{kj}\left(t\right)+c_{1}r_{1}\left(p{\text{best}}_{kj}\left(t\right)-x_{kj}\left(t\right)\right)+c_{2}r_{2}\left(g{\text{best}}_{kj}\left(t\right)-x_{kj}\left(t\right)\right),\\ x_{kj}\left(t+1\right)=v_{kj}\left(t+1\right)+x_{kj}\left(t\right), \end{array}$$

where *j*(*j*=1,…, *n*), *t* is the generation, *w* is the inertia weight which is used to balance local and global search, *c*_1_ and *c*_2_ are two learning factors from the personal and global best particles, which are called the acceleration coefficients too, and *r*_1_ and *r*_2_ are elements from two uniform random sequences in the range of [0,1].

### 2.3. *θ*-DEA

The newly proposed evolutionary algorithm (*θ*-DEA) [28] is based on a new dominance relation *θ*-dominance, which improves the convergence of NSGA-III [10] by referring to the procedure of the MOEA/D [4] algorithm, and still inherits the advantage of NSGA-III in maintaining diversity. In the *θ*-dominance, the solution is divided into different groups by giving a set of well-distributed reference points, where each group is represented by a reference point. A fitness function similar to PBI is defined to select a set of optimal solutions. The algorithm framework of *θ*-DEA [28] is summarized as follows.

Firstly, a group of uniformly distributed reference points is generated in the objective space and population *P* is initialized. Ideal point *z*^*∗*^ and the nadir point *z*^nad^ can be easily obtained. Then, the recombination operator is used to recombine *P* to generate offspring population, which is combined with the current population *P* to compose a new population *S*. Next, a new population *Q* is obtained based on the Pareto nondominated level of the population *S*. The population *Q* is normalized with the help of *z*^*∗*^ and *z*^nad^, and the population *Q* is clustered by the clustering operator, where each reference point represented a cluster. Finally, the nondominated sorting based on *θ*-dominance is employed to classify *Q* into different *θ*-dominance levels. The algorithm selects the solutions according to the different levels of *θ*-dominance until the termination condition is reached.

### 2.4. Some Current Multi/Many-Objective Particle Swarm Optimization

Particle swarm optimization is often used to solve multiobjective optimization problems because of its fast convergence and easy implementation. At present, there are many research literatures on multiobjective particle swarm optimization, such as NMPSO [34], MPSO-D [6], and D^2^MOPSO [21]. The existing multiobjective particle swarm optimization algorithms can be generally summarized into the following types. The first type is formed by the multiobjective particle swarm optimization algorithm dominated by Pareto, which determines the personal best and global best particles based on the Pareto ranking. Through Pareto ranking and finite iteration updating of the nondominance solution, the algorithm exploits and explores the global best particles that are close to the entire true PF. The classic multiobjective optimization problems include MOPSO [22], OMOPSO [23], SMPSO [35], CMOPSO [27], and AMOPSO [36]. The second category adopts the decomposition method, which decomposes the MOP into a group of single-objective optimization problems and solves each single objective optimization problem directly by PSO. This approach has been proved to be effective in solving complex MOPs, and its representative algorithms are dMOPSO [29], D^2^DMOPSO [21], MOPSO/D [37], MPSO/D [6], AgMOPSO [7], and MMOPSO [24]. We select a few of the latest algorithms in these algorithms for a brief overview.

MPSO-D [6] employs a set of direction vectors to decompose the target space into a set of subspaces and ensures that each subregion has a solution. Moreover, the crowding distance and adjacent particles are used to determine the global best historical position of the particles.

MMOPSO [24] takes the decomposition approach to transform the multiobjective optimization problem into a group of aggregation problems and adopts the double search strategy to improve the velocity of updating particles. The weakness of PSO search can be effectively improved by evolutionary search on the nondominant solutions stored in external archives.

COMPSO [27] adopts Pareto dominance and competition-based learning strategy which are used to update particles, where each particle learns from the winner of each pair completion. However, competition is only between elite particles selected in current population, and there is no external archive to save global and personal best particles.

AgMOPSO [38] also transforms the multiobjective optimization problems into a set of aggregation subproblems by the decomposition method and optimizes them with the allocated particles. Archive-guided velocity update method is employed to guide the swarm for exploration, and the Pbest, lbest, and gbest particles are selected from the external archive, which is evolved by the immune evolution strategy.

In addition, there are recently proposed MOPSO methods based on indicators, reference points, and balanceable fitness estimation, such as NMPSO [34], MaOPSO [39], and R2-MOPSO [40], which are used to solve high-dimensional multiobjection optimization problems. NMPSO [34] uses a balanced fitness estimation method, which combines convergence and diversity distance to solve many-objective optimization problems. In order to enhance the performance of the algorithm, evolutionary search is used in external archives and a new PSO speed update equation is applied. The algorithm MaOPSO [39] adopts a set of reference points that are dynamically determined based on the search process and imposes the necessary selection pressure on the algorithm to make it converge to the true PF, while maintaining the diversity of the PF. R2-MOPSO [40] combines R2 performance metrics with particle swarm optimization and guides the search through a well-designed interactive process for solving many-objective optimization problems.

## 3. The Proposed Algorithm

In this section, the proposed algorithm CMaPSO is mainly described in detail, since the competitive swarm operator can achieve a better balance between convergence and diversity than the traditional particle swarm optimization algorithm. In this paper, the particle swarm optimization algorithm based on the competitive mechanism is combined with another environment selection mechanism, which is different from the algorithm CMOPSO [27]. In order to improve the convergence and diversity of the proposed algorithm in solving many-objective optimization problems, the recently proposed *θ*-dominance is adopted to further enhance the selection pressure for nondominated solutions. However, experiments show that simply using *θ*-dominance can cause the algorithm to perform poorly on some degenerate problems. Therefore, a reference points regeneration strategy is used to improve this deficiency. We will describe the main components of the algorithm CMaPSO, including competitive mechanism for velocity update and position, environmental selection based on extreme points, and *θ*-dominance are described. Finally, we will give the complete framework of the algorithm CMaPSO.

### 3.1. Competitive Mechanism for Velocity Update and Position

Similar to the algorithm CMOPSO, the population is sorted by nondominated sorting and crowding distance ranking, but the difference is that according to the current population size, different numbers of outstanding individuals are obtained from the sorted population to form a competitive group. Crowding distance [41] is an index to describe the degree of crowding between individuals in the same nondominated front and their neighbors. A pair of particles are randomly selected from the current competitive group, where the winners will be used to guide the moving directions of particles in the current swarm.

**Figure 1:**
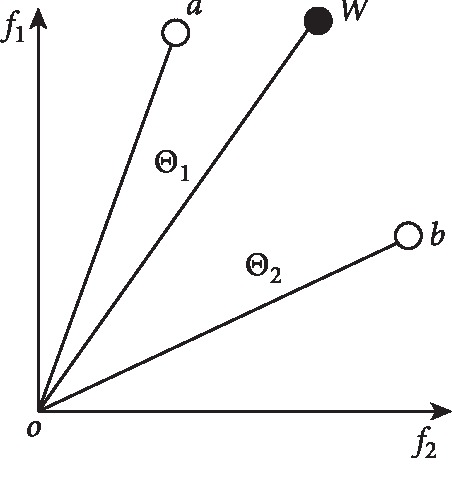
An example of elite particle generation.

The whole process of how to generate elite particles is called competition mechanism.  Figure 1 shows the process of generating elite particles. For each pairwise competition, given a particle *w* in the swarm, two elite particles *a* and *b* are randomly selected from the competitive group. The elite particle with a smaller angle wins the competition after the angles between *a*, *b*, and *w* are calculated, respectively. It is easy to find from the figure that the angle *θ*_1_ of *a* to *w* is significantly smaller than the angle *θ*_2_ of *b* to *w*, so *a* as an elite particle guides the evolution of the population.

The updated velocity ${\bar{v}}_{i}$ and position ${\bar{p}}_{i}$ of the *i*th particle are calculated using the following equations as suggested in the competitive swarm optimizer:

(6) $$\begin{array}{c} {\bar{v}}_{i}=c_{1}R_{1}v_{i}+c_{2}R_{2}\left(p_{w}-p_{i}\right), \end{array}$$

(7) $$\begin{array}{c} {\bar{p}}_{i}=p_{i}+{\bar{v}}_{i}, \end{array}$$

where *R*_1_,  *R*_2_ ∈ [0,1] are two randomly generated vectors and *p*_*w*_ is the position of the winner, and *c*_1_ and *c*_2_ are learning factors. The pseudocode for modified competition mechanism-based learning strategy is described in Algorithm 1. Finally, the polynomial mutation are widely adopted in the multiobjective optimization algorithm [24, 27] is used to mutate the population to enhance the diversity. The expression of polynomial variation is as follows:

(8) $$\begin{array}{c} v_{k}^{2}=v_{k}+\delta \times \left(u_{k}-l_{k}\right), \end{array}$$

where

(9) $$\begin{array}{c} \delta =\left\{\begin{array}{cc} 2u+\left(1-2u\right){\left(1-\delta_{1}\right)}^{1/\left(\eta_{m}+1\right)}, & \text{if}u\leq 0.5,\\ 1-{\left[2\left(1-u\right)+\left(u-0.5\right){\left(1-\delta_{2}\right)}^{\eta_{m}+1}\right]}^{1/\left(\eta_{m}+1\right)}, & \text{if}u>0.5, \end{array}\right. \end{array}$$

with *δ*_1_=(*v*_*k*_ − *l*_*k*_)/(*u*_*k*_ − *l*_*k*_), *δ*_2_=(*u*_*k*_ − *v*_*k*_)/(*u*_*k*_ − *l*_*k*_), *u* is a random number in [0,1], *η*_*m*_ is the distribution index, and *v*_*k*_ is a parent individual.

### 3.2. Generation of Reference Points

In this paper, *θ*-dominance ordering is adopted to increase the selection pressure of the algorithm, and the selection of nondominated solution is implemented by the guidance of reference points. Therefore, it is necessary to introduce the generation of reference points. Reference point methods are often used to improve MOEAs' diversity and uniformity capacity in solving the multi/many-objective optimization problem, such as NSGA-III [10] and *θ*-EDA [28]. These algorithms first generate a set of predefined or user-preferred reference points based on the decomposition. This paper adopts a two-layer (boundary and inside layers) approach [10, 28], which uses the systematic approach to generate two reference direction sets: one set on the boundary layer and the other on the inside layer. Suppose the divisions of boundary and inner layers is *H*_1_ and *H*_2_, respectively, then number of reference points is computed as follows:

(10) $$\begin{array}{c} P=\left(\begin{array}{c} H_{1}+m-1\\ H_{1} \end{array}\right)+\left(\begin{array}{c} H_{2}+m-1\\ H_{2} \end{array}\right), \end{array}$$

where *m* is the dimension of objective space and *H*_1_ and *H*_2_ are user-defined integers.

### 3.3. *θ*-Dominance Sorting

*θ*-dominance adopts the PBI (penalty-based boundary intersection) method of MOEA/D [4]. And the PBI scalar function is defined as follows:

(11) $$\begin{array}{c} \underset{x\in \Omega }{\min}g^{\text{PBI}}\left(\left.x\right|\lambda ,z^{*}\right)=d_{1}+\theta d_{2}, \end{array}$$

where

(12) $$\begin{array}{c} d_{1}=\frac{\left\|{\left(F\left(x\right)-z^{*}\right)}^{T}\lambda \right\|}{\left\|\lambda \right\|}, \end{array}$$

(13) $$\begin{array}{c} d_{2}=\left\|\left(F\left(x\right)-z^{*}\right)-d_{1}\frac{\lambda }{\left\|\lambda \right\|}\right\|, \end{array}$$

*θ* > 0 is a preset penalty parameter and generally set to *θ*=5. *z*^*∗*^=(*z*_1_^*∗*^, *z*_2_^*∗*^,…, *z*_*m*_^*∗*^) is the reference point, i.e., *z*_*i*_^*∗*^=min{*f*_*i*_(*x*)|*x* ∈ *Ω*}  for each *i*=1,2,…, *m*.

The normalized population is clustered by PBI scalar function, and the *θ*-dominance ordering is performed according to the value of PBI, where the extreme solutions are controlled by the size of *θ*. The definition of *θ*-dominance is as follows: given two solutions *x*, *y* ∈ *S*_*t*_, *x* is said to *θ*-dominate *y*, denoted by *x*≺_*θ*_*y*, iff *x* ∈ *C*_*i*_, *y* ∈ *C*_*i*_ and *F*_*i*_(*x*) < *F*_*i*_(*y*), where *i* ∈ {1,  2,…, *N*}. The purpose of *θ*-dominance is to obtain a set of nondominated solutions nearest to PF and well distributed along PF. The pseudocode of the *θ*-dominance sorting process is shown in Algorithm 2.

### 3.4. The Reference Point Regeneration Strategy

The uniformly distributed reference point is based on the general assumption that PF has a regular geometric structure, that is, smooth, continuous, and well distributed. However, there are various multiobjective optimization problems in practical applications. The geometric structure of PF may be very irregular, such as discontinuous, degenerate, and multimodal. For these problems, if uniformly distributed reference vectors are still employed and some reference vectors may not be associated with individuals, then the density of Pareto optimal solutions is insufficient. Based on such problems, we will adopt the reference vector regeneration strategy proposed in RVEA [11]. This strategy mainly regenerates reference points and replaces those that are not used and wasted. The pseudocode of the reference points regeneration strategy is shown in Algorithm 3.

### 3.5. Environmental Selection Based on Extreme Points

In this paper, we discuss the extreme points, which mainly refer to the extreme solutions in the normalized solution set. Let *X*=(*x*_1_, *x*_2_,…, *x*_*M*_) be a solution in the normalized solution set. If solution *X* has a component *x*_*i*_, *i*=1,2,…, *M*, which is the maximum or minimum value of the corresponding position component in all solutions, then this solution is defined as an extreme solution here. Firstly, the extreme solutions are selected to enter the archive. Then, the angle between the unselected solution and the selected solutions are calculated, and the solution entering the archive is selected by the size of angle. The angle between solutions *x*_*i*_ and *y*_*j*_ is defined as follows:

(14) $$\begin{array}{c} \text{angle}\left(x_{i},y_{j}\right)≜\arccos\left|\frac{F^{\prime }\left(x_{i}\right)\cdot F^{\prime }\left(y_{j}\right)}{x_{i}\cdot y_{j}}\right|. \end{array}$$

Obviously, angle(*x*_*i*_, *y*_*j*_) ∈ [0, *π*/2].

**Figure 2:**
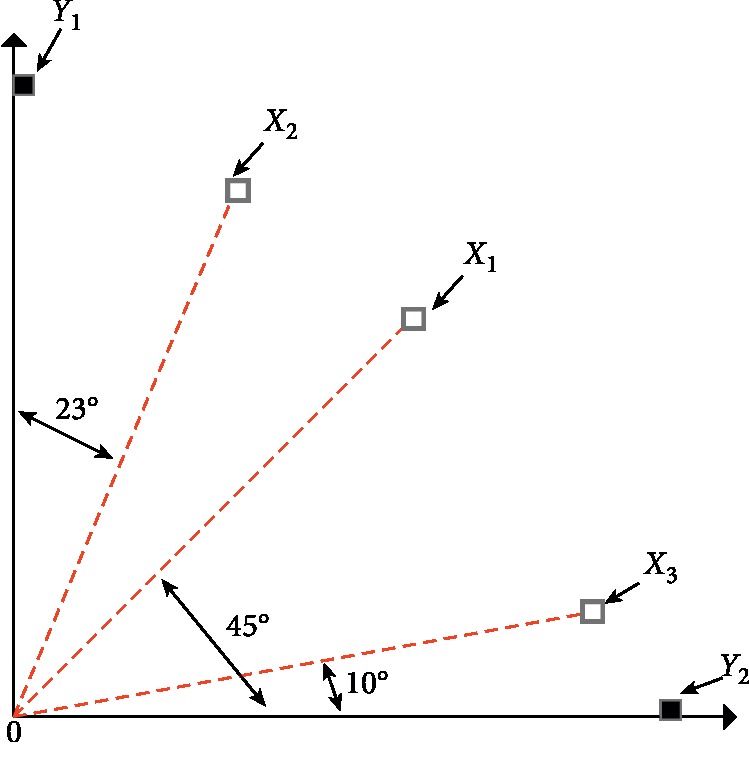
Illustration of individual selection by angle size.

The process of archives updating based on extreme points is illustrated in Figure 2. Suppose there is a population in the objective space, including five individuals *X*_1_, *X*_2_, *X*_3_, *Y*_1_, and *Y*_2_. Firstly, the population is normalized, and the two extreme points *Y*_1_ and *Y*_2_ are selected by calculation. Then, the angles between *X*_1_, *X*_2_, and *X*_3_ and *Y*_1_ and *Y*_2_ are calculated, respectively, and the maximum is obtained after minimizing the results. *X*_1_ is first selected to enter into the optimal population, and in this way, *X*_2_ and *X*_3_ are sequentially selected into the optimal population. It can be seen from the individual selection process that the individual who is away from the two extreme points and in the middle of the two extreme points is preferentially selected, which can ensure the diversity and uniformity of the optimal population.  Algorithm 4 shows pseudocode for archives updating based on extreme points.

### 3.6. The Complete CMaPSO Algorithm

The complete framework of the proposed CMaPSO method is described in Algorithm 5. Firstly, the population is initialized, and the corresponding population number is obtained. A set of uniformly distributed reference points K are generated using equation (8). Then, the external archives are updated with the existing information. In the while loop, the competition mechanism of PSO algorithm (Algorithm 1) is used to update the population *P* to obtain a new population *P*. The next step is to update the external archives with the new population *P*, and the elite population *A* is obtained. In order to avoid the algorithm falling into local optimum, the genetic operator is used to cross and mutate external archives *A* so that the information between elite individuals can be fully exchanged, and new population *S* can be generated. The external archives are updated by combining the new population S and elite population *A*, and the new elite population *A* is gained. Finally, under certain conditions, the reference point regeneration strategy is adopted to obtain new reference points to replace the previous according to the distribution of individuals in archives *A*. The algorithm continues the while-loop until the termination condition is satisfied.

### 3.7. Computational Complexity of Proposed Algorithm CMaPSO

We consider the main steps of the proposed algorithms and analyzed the algorithm complexity of the algorithm CMaPSO. The algorithm CMaPSO is mainly composed of learning based on competition mechanism, updating elite archives, *θ*-dominance sorting, and reference points regeneration strategy. The time complexity of these main parts is *O*(*mN*^2^) in the worst case. Therefore, the overall worst-case time complexity of a generation in CMaPSO is *O*(*mN*^2^).

## 4. Experimental Studies

This section is devoted to the research of simulation experiments by selecting appropriate comparative algorithms, standard benchmark problems, performance metrics, etc. The performance of the proposed algorithm CMaPSO is evaluated by comparison experiment results. The background of simulation experiment is introduced, including standard benchmark problems, performance metrics, experimental environment, and some parameters setting. In order to evaluate the performance of CMaPSO, the recently published advanced algorithms are compared with the algorithm. Four recently proposed multi/many-objective particle swarm optimization algorithms, such as CMOPSO [27], MMOPSO [24], MPSOD [6], and dMOPSO [29] and four recently proposed many-objective evolutionary optimization algorithms, such as NSGA-III [10], MOBIII [8], RPEA [30], and MyODEMR [31] are employed to validate the performance of the proposed algorithm. In the simulation experiments, we use the parameters proposed in the original paper to set the comparative algorithms.

### 4.1. Standard Benchmark Problems

In order to verify the performance of the proposed algorithm CMaPSO, a total of 25 benchmark MOPs with 3, 5, 8, 10, and 15 objectives from two test suits are used to evaluate the performance of the proposed algorithm CMaPSO, including DTLZ [42], WFG [43], and UF [44], where DTLZ1 to DTLZ7, WFG1 to WFG9, and UF1 to UF9 are considered. The Pareto front characteristics of test problems adopted in simulation experiments are summarized, as shown in Table 1. The mathematical expressions of the test sets DTLZ, WFG, and UF are presented in Supplementary materials (available ([Supplementary-material supplementary-material-1])).

We have adopted the proposals for setting decision variables of test suites DTLZ and WFG in [34, 45]. For DTLZ1 to DTLZ7, the number of decision variables is set as *d*=*m*+*k* − 1, where *n* is the number of decision variables, *m* is the number of objective, and *k* is the fixed parameter. For WFG1 to WFG9, the number of decision variables is set as *d*=*k*+*l*, where *n* is the number of decision variables, *k* is set as 2 *∗*(*m* − 1), *m* is the objective number, and *l* is the fixed parameter. The number of objectives and decision variables in the UF test set are arranged according to the recommendations in [44], where the test functions UF1 to UF7 are with the objective number *m* = 2, the number of decision scalars *d* = 30, and UF8 to UF9 with the objective number *m* = 3 and *d* = 30. The detailed setting information is shown in Table 2.

### 4.2. Parameter Setting

In the simulation experiment of this paper, different population numbers are set for different dimensions, and the number of evaluations is uniformly set to 150000, as shown in Table 3. For each test case, all employed algorithms run independently for 30 times on a PC with Intel Core i7-6500u @ 2.50 GHz dual-core CPU and Microsoft Windows 10 operating system. The simulation experiment was carried out on the recently proposed PlatEMO-2.0 [46]. The average value and standard deviation of the simulated data of all the comparison algorithms are compared to illustrate the performance of the algorithm, where the best performance value on each test instance is highlighted with a bold background. Moreover, the Wilcoxon rank sum test is adopted at a significance level of 0.05, where the symbols “+,” “−,” and “=” indicate that the result is significantly better, significantly worse, and statistically similar to that obtained by the proposed algorithm.

### 4.3. Performance Metrics

Performance evaluation indicators are used to measure the convergence and distribution performance of the solution set obtained by the algorithm. The commonly used performance evaluation indicators are as follows.

#### 4.3.1. Generation Distance

Generation distance (GD) [47] is used to evaluate the convergence performance of the algorithm. The smaller the GD value of the solution set obtained by an algorithm, the better the convergence performance of the algorithm. In particular, when GD = 0, all the optimal solutions obtained by the algorithm are on the true Pareto front.

#### 4.3.2. Spacing

Spacing (SP) [47] is used to reflect the uniformity of the solution set distribution obtained by the algorithm. The smaller the SP value, the more uniform the solution set distribution.

#### 4.3.3. Inverted Generational Distance

The inverted generational distance (IGD) [48] is one of the most widely used indicators. It could provide comprehensive information about convergence and distribution performance of the algorithm by calculating the minimum distance sum between each point on the true PF and the calculated individual set. The smaller the IGD value, the better the quality of the solution set obtained by the algorithm. Particularly, if IGD is equal to 0, the obtained PF contains every point of the true PF.

In this paper, IGD is employed to evaluate the performance of the algorithm. Let *S* be a uniformly distributed subset selected from the true *PF* and *S*′ is the optimal Pareto solution set obtained by the current algorithm. The IGD is defined as follows:

(15) $$\begin{array}{c} \text{IGD}\left(S,S^{\prime }\right)=\frac{\sum_{i=1}^{\left|S\right|}d\left(S,S^{\prime }\right)}{\left|S\right|}, \end{array}$$

where |*S*| returns the number of solutions in set *S* and *d*(*S*, *S*′) computes the minimum Euclidean distance from *S*_*i*_ ∈ *S* to the solutions of *S*′ in the objective space.

In addition, to intuitively quantify the overall performance of the algorithm on different test problems, the performance score method is introduced. Suppose there are ℒ algorithms *A*_1_, *A*_2_,…, *A*_ℒ_, if algorithm *A*_*i*_ is significantly better than algorithm *A*_*j*_ according to IGD, *δ*_*ij*_ is set as 1, and 0 otherwise. Then, for each algorithm *A*_*i*_, the performance score *P*(*A*_*i*_) is computed by the following equation:

(16) $$\begin{array}{c} P\left(A_{i}\right)=\sum_{j=1,j\neq i}^{ℒ}\delta_{ij}. \end{array}$$

The performance score indicates how many other algorithms are better than the corresponding algorithm on the test problems, and smaller values represent better performance than other algorithms.

### 4.4. Performance Comparison between CMaPSO and Four Multi/Many-Objective Particle Swarm Optimization Algorithms

**Figure 3:**
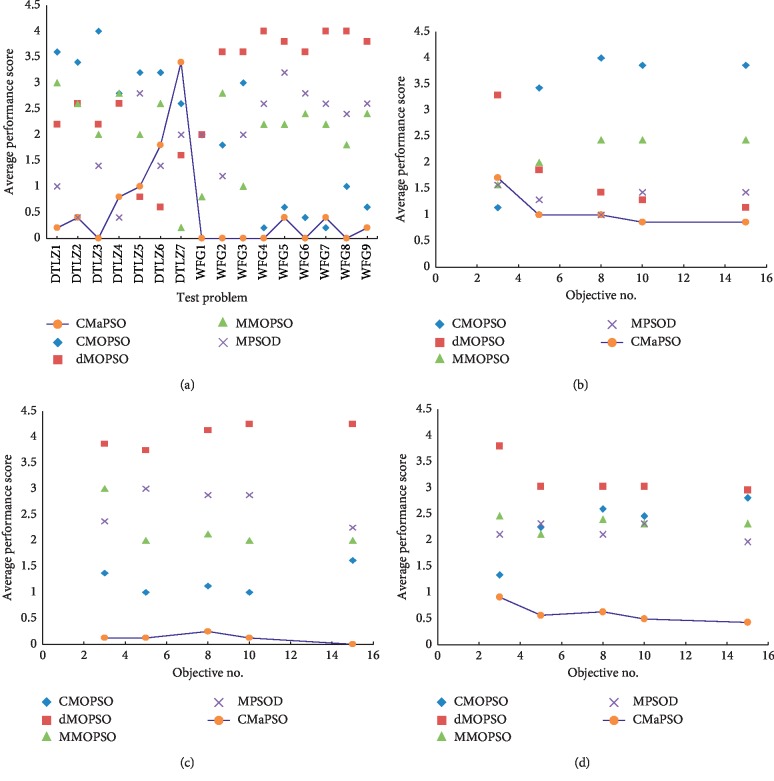
Average IGD performance scores for different objectives and different test instances. (a) Average performance scores over all dimensions for different test cases. (b) Average performance scores of the test instances DTLZ for different number of objectives. (c) Average performance scores of the test instances WFG for different number of objectives. (d) Average performance scores over all test instances for different number of objectives.

**Figure 4:**
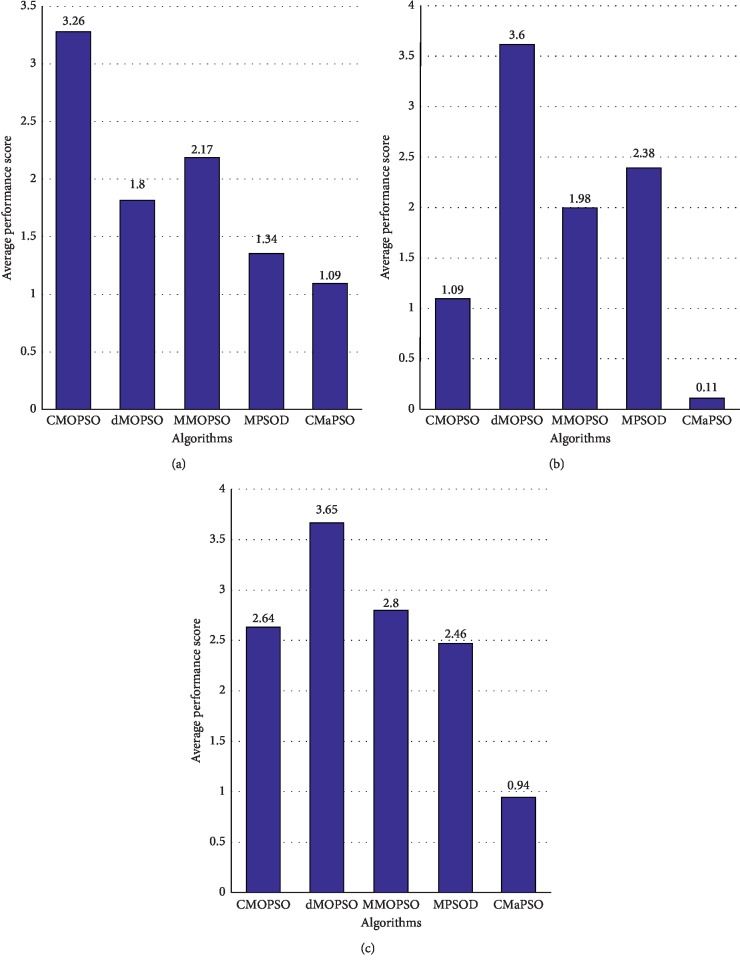
All comparison algorithms have average performance scores for IGD on (a) DTLZ, (b) WFG, and (c) two test suites.

This section we compare the proposed algorithm CMaPSO with four recently published multi/many-objective particle swarm optimization algorithms CMOPSO, MMOPSO, dMOPSO, and MPSOD on the 3, 5, 8, 10, and 15 objectives of DTLZ and WFG test suites, respectively. The comprehensive performance index IGD is used to evaluate the simulation results, and the experimental results can illustrate the performance of the algorithm on different test cases. Performance score is used to intuitively illustrate the performance of each algorithm. Tables 4 and 5 show the simulation results of the proposed algorithms and other comparative algorithms on DTLZ and WFG test suites, where the experimental results are reported by the mean and standard deviation of IGD values. Tables 6 and 7 lists the number of the best, better, similar, and worse performance of CMaPSO and comparative algorithms on DTLZ and WFG, respectively.  Table 8 summarizes the experimental results in Tables 6 and 7 to illustrate the total significance test of each comparative algorithms on all test issues.  Figure 3 depicts the trend of the average IGD performance scores of all comparative algorithms for different objectives and different test instances and links the trends of the proposed algorithm with blue polylines.  Figure 4 shows the average performance scores of all comparative algorithms on DTLZ and WFG test suites using histogram.

Table 4 summarizes the simulation results of five multi/many-objective particle swarm optimization algorithms on the test set DTLZ, and the best value for each test case is highlighted with a bold background. From the table, it can be seen intuitively that the number of the best values of the proposed algorithm CMaPSO on test cases DTLZ1, DTLZ2, DTLZ3, and DTLZ4 accounts for the majority. The algorithm MMOPSO performs best on test case DTLZ7. The algorithm CMaPSO does not perform as well as the algorithm dMOPSO on the DTLZ5 and DTLZ6 test cases, and the number of best values of the algorithm dMOPSO on these two test cases is ahead of other algorithms. From Figure 3(a), the proposed algorithm CMaPSO has the best comprehensive IGD performance score on test cases DTLZ1, DTLZ2, and DTLZ3. In addition, we can find that the algorithm MPSOD has the best comprehensive IGD performance score on DTLZ2 and DTLZ4. dMOPSO achieves the best on DTLZ5 and DTLZ6. MMOPSO gains the best on DTLZ7. CMOPSO does not obtain the best performance on any test case of DTLZ. From Figure 3(b), it is obvious that the proposed algorithm CMaPSO has the best IGD performance score on the 5, 8, 10, and 15 objectives of DTLZ in all comparative algorithms. And the performance score of the proposed algorithm on 3 objectives is not as good as the algorithm CMOPSO. The histogram of Figure 4(a) synthetically reflects IGD performance scores of all comparative algorithms on the DTLZ, which can effectively evaluate the performance of each algorithm. The proposed algorithm CMaPSO obtains the best IGD performance score.

Table 6 summarizes the significance test results of the proposed algorithm CMaPSO and the comparative algorithms on the test set DTLZ. There are a total of 35 test cases on the test set DTLZ. The proposed algorithm CMaPSO has 15 test cases ranking first, accounting for 42.86% of the total. The comparative algorithms CMOPSO, dMOPSO, MMOPSO, and MPSOD obtain 3, 8, 4, and 5 test cases ranking first, accounting for 8.57%, 22.86%, 11.43%, and 14.29% of the total, respectively. The algorithm MPSOD and dMOPSO perform best in the four employed comparative algorithms. From the results of the abovementioned data analysis, it can be concluded that the performance of the algorithm CMaPSO is better than the comparative algorithms on the DTLZ test suite.

Table 5 presents the simulation results of the five comparative algorithms on the test set WFG, and the best values of each test case is also marked in a bold background. Obviously, the algorithm CMaPSO gets the best performance on most test cases of WFG. The performance of the comparative algorithms on WFG is almost common, which is inferior to the proposed algorithm.

Figure 3(a) intuitively shows that CMaPSO obtains the best performance scores on test cases WFG1, WFG2, WFG3, WFG4, WFG5, WFG6, WFG8, and WFG9, but does not include the test case WFG7. The main reason is that although the algorithm CMaPSO achieves the best performance on the 3, 5, and 15 objectives of WFG7, the performance is not satisfactory on the 8 and 10 objectives, and the average performance score mainly emphasizes the average performance of the algorithm on test cases. Noticed that the algorithm CMOPSO only gets two the best performance on test case of WFG7, its average performance score is the best. As shown in Figure 3(c), the average performance score of the algorithm CMaPSO on the 3, 5, 8, 10, and 15 objectives of the WFG is better than the comparative algorithms adopted. The average performance score of the comparative algorithms on different test cases of WFG are obtained by comprehensive calculation, and the histogram is used to intuitively represent the scores. From Figure 4(b), algorithm CMaPSO obtained 0.94 points, which is the best score among all the comparative algorithms. Other algorithms CMOPSO, dMOPSO, MMOPSO, and MPSOD got 2.64, 3.65, 2.8, and 2.46 points, respectively.

In this paper, all the comparative algorithms are simulated on 45 test instances of WFG. From Table 7, the significance test of algorithm CMaPSO ranks first on 34 test cases, accounting for 75.56% of the total. The comparative algorithms CMOPSO, dMOPSO, MMOPSO, and MPSOD obtains 10, 0, 1, and 0 test cases ranking first, accounting for 22.22%, 0%, 2.22%, and 0% of the total, respectively. Among the four comparative algorithms, the algorithm CMOPSO has the best performance on WFG. There are 5 test cases whose significance test is better than the algorithm CMaPSO on the WFG test problems, 12 test cases is similar to the CMaPSO, and 28 test cases is obviously inferior to the algorithm.

**Figure 5:**
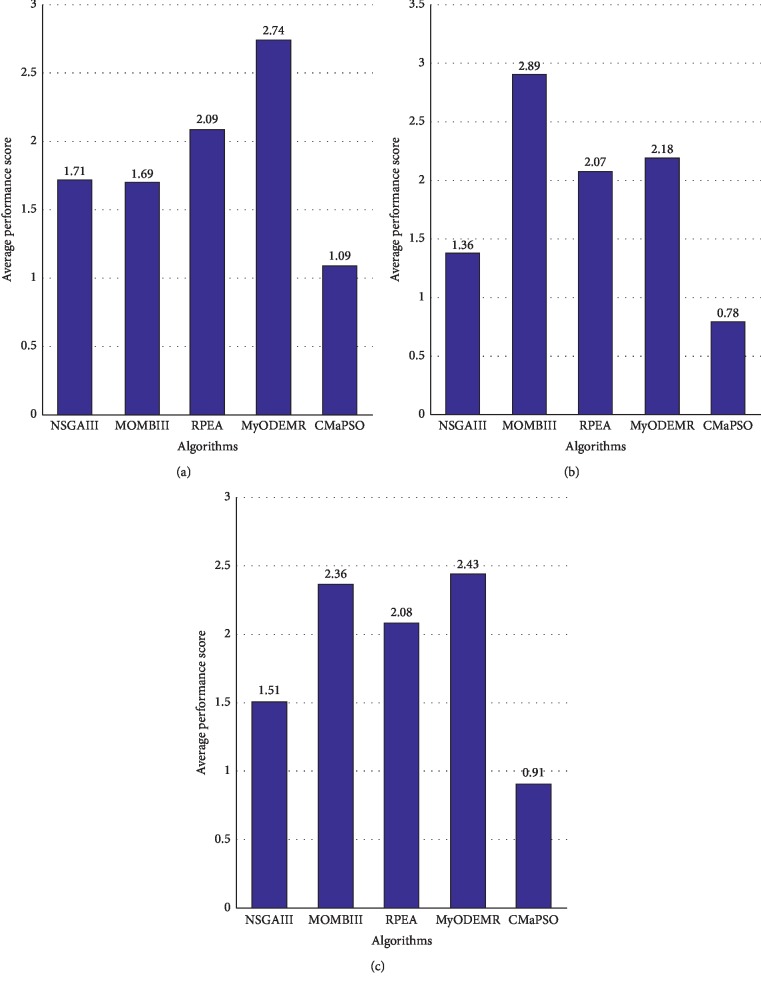
All comparison algorithms have average performance scores for IGD on (a) DTLZ, (b) WFG, and (c) two test suites.

We have analyzed the performance of all the comparative algorithms on the DTLZ and WFG test suites, respectively. Then, we combine the two test suites and make a comprehensive analysis of the performance of all the comparative algorithms. From Table 8, the number of significant test results ranked first obtained by CMaPSO, CMOPSO, dMOPSO, MMOPSO, and MPSOD on all test cases are 49, 13, 8, 5, and 5, accounting for 61.25%, 28.89%, 10%, 6.25%, and 6.25% of the total, respectively. As shown in Figure 3(d), the average performance score of CMaPSO is better than the other comparative algorithms on the 3, 5, 8, 10, and 15 objectives of the test instances. The algorithm CMaPSO achieves better performance than the algorithm CMOPSO which also adopts competitive mechanism. It can also be effectively illustrated from histogram Figure 4(c). Figures 5(a)–5(e) illustrates the approximate Pareto front obtained by five comparative algorithms on the 15 objectives of DTLZ1. By comparing these figures, the result obtained by CMaPSO is the closest to the true PF in the comparative algorithms.

### 4.5. Performance Comparison between CMaPSO and Four Advanced Many-Objective Evolutionary Optimization Algorithms

**Figure 6:**
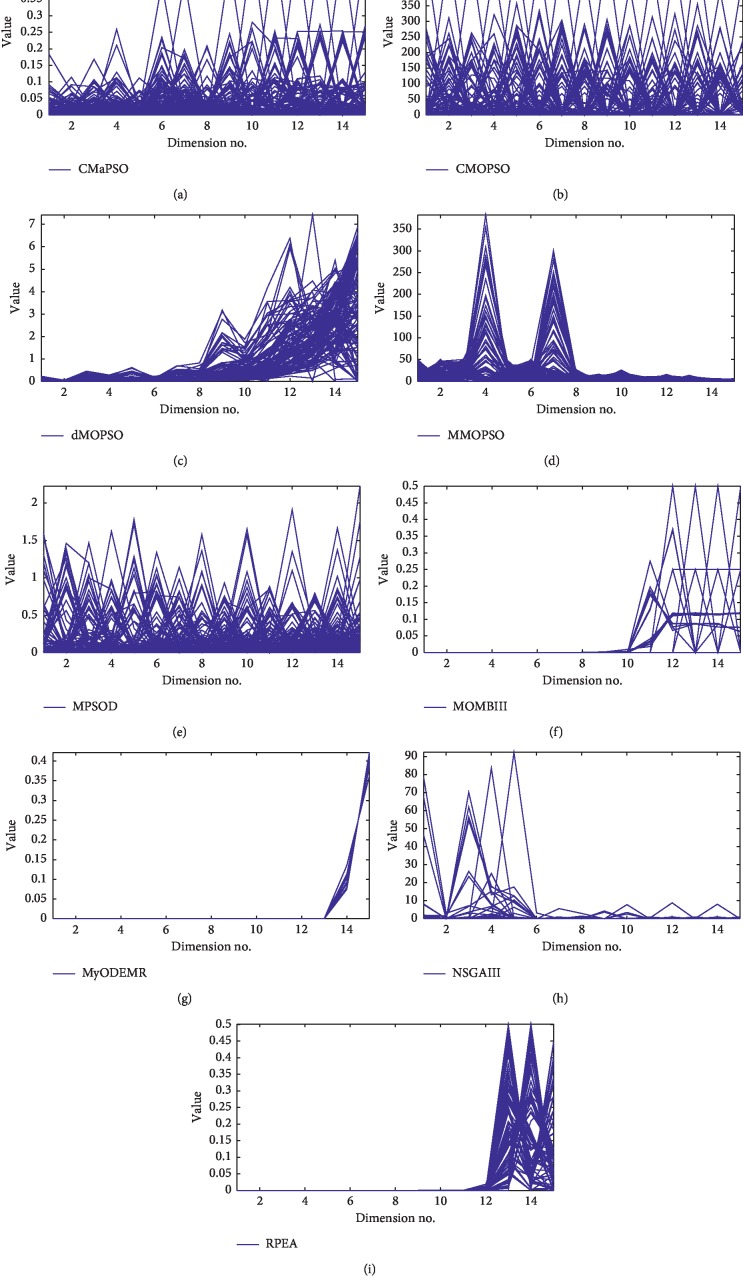
The approximate Pareto fronts obtained by (a) CMaPSO, (b) CMOPSO, (c) dMOPSO, (d) MMOPSO, (e) MPSOD, (f) MOBIII, (g) MyODEMR, (h) NSGA-III, and (i) RPEA on DTLZ1 (15-objecive) are shown by a parallel coordinate.

**Figure 7:**
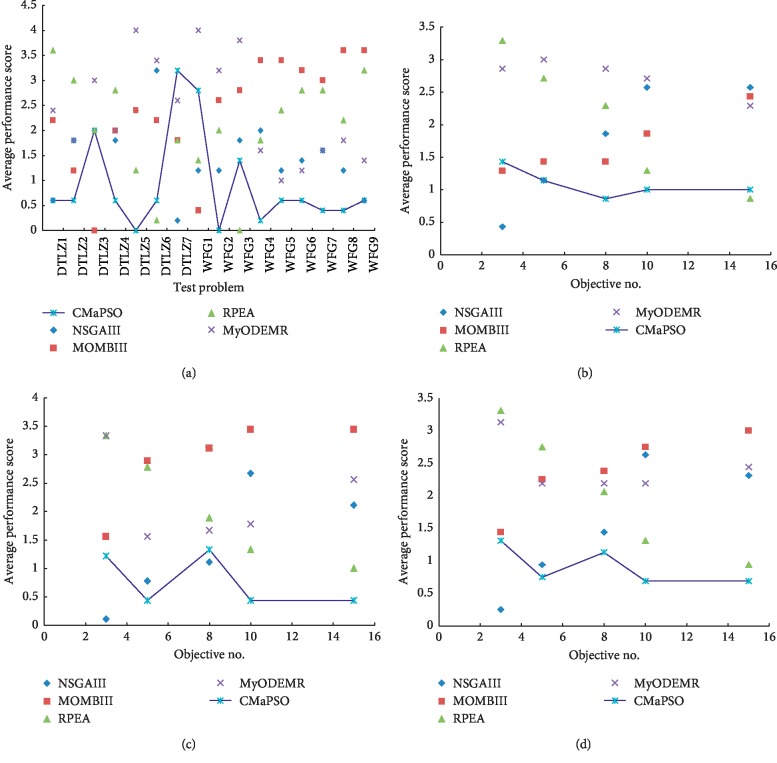
Average IGD performance scores for different objectives and different test instances. (a) Average performance scores over all dimensions for different test cases. (b) Average performance scores of the test instances DTLZ for different number of objectives. (c) Average performance scores of the test instances WFG for different number of objectives. (d) Average performance scores over all test instances for different number of objectives.

In this section, the performance of the proposed algorithm CMaPSO is verified by simulation experiments, which is compared with four recent evolutionary algorithms on DTLZ and WFG test suites. The comprehensive performance metric IGD is adopted to illustrate the experimental results. Tables 9 and 10 summarize the experimental results. Tables 11 and 12 statistics the number of the best, better, similar, and worse performance of CMaPSO and comparative algorithms on DTLZ and WFG, respectively.  Table 13 summarizes the experimental results in Tables 11 and 12 to illustrate the total significance test of each comparative algorithms on all test issues.  Figure 6 illustrates the average performance scores of all comparative algorithms for different objectives and test problems.  Figure 7 shows the average performance scores of all comparative algorithms on DTLZ and WFG and the two test suites, respectively.

From Table 9, the algorithm CMaPSO has the best performance on DTLZ1, DTLZ4, and DTLZ5 test problems and achieves the best performance on 8, 10, and 15 objectives. The proposed algorithm does not get most of the best performance on the 3, 5, 8, 10, and 15 objectives of the test problem DTLZ2 and only performs best on the 5 and 15 objectives, but it still achieves the best performance by a slight advantage compared with the comparative algorithms. From Figure 6(a), the average performance score of CMaPSO on test cases DTLZ1, DTLZ2, DTLZ4, and DTLZ5 is the best. And From Figure 6(b), it is obviously observed that the best average performance score of CMaPSO for different objective numbers on test suite DTLZ is 5, 8, and 10 objectives. The comparative algorithm NSGA-III has the best performance on DTLZ7 test problem and obtains the best performance on 3, 5, 8, and 10 objectives, respectively. Notice that the proposed algorithm has the worst performance on DTLZ7 compared with the other four comparative algorithms. The comparative algorithms NSGA-III and MOBIIII achieve the same number of best performances on DTLZ3, where NSGA-III obtains the best performance on 3 and 5 objectives, while MOBIII gains the best performance on the 8 and 10 objectives of the test problem DTLZ3. The algorithm RPEA gets the best performance on the 5, 8, and 15 objectives of the test problem DTLZ6.

Table 11 summarizes the number of the best, better, similar, and worse performance of CMaPSO and comparative algorithms on DTLZ. From Table 9, there are a total of 35 test cases. And the proposed algorithm has 16 test cases ranking first, accounting for 45.7% of the total from Table 11. In addition, it can be clearly observed from Figure 7(a) that the proposed algorithm has the best average performance score for different objective numbers on the test suite DTLZ. The comparative algorithms NSGA-III, MOBIII, RPEA, and MyODEMR achieves 9, 3, 5, and 2 test cases ranking first, accounting for 25.7%, 8.5%, 14.2%, and 5.7% of the total, respectively. The algorithm MOBIII performs best in all the comparative algorithms. A total of 13 test cases are significantly better than the algorithm CMaPSO on the DTLZ, but 21 test instances are obviously worse than CMaPSO. From the results of data analysis, it can be concluded that the performance of the algorithm CMaPSO is better than that the comparative algorithms on the DTLZ test suite.

From Table 10, the algorithm CMaPSO has the best performance on WFG2, WFG4, WFG5, WFG6, WFG7, and WFG9 test problems and basically achieves the best performance on 5, 10, and 15 objectives. The proposed algorithm and the comparative algorithm NSGA-III have similar performance on WFG8 test problem, where NSGA-III gets the best performance on 3 and 5 objectives, while CMaPSO has the best performance on 10 and 15 objectives. From Figure 6(a), it can be intuitively observed that the proposed algorithm has the best performance scores on the test cases WFG2, WFG4, WFG5, WFG6, WFG7, WFG8, and WFG9. Moreover, from Figure 6(c), the average performance score of the proposed algorithm is the best on the 5, 10, and 15 objectives of WFG test suite. The performance of RPEA ranks first on the test problem WFG3, which is superior to other algorithms. Algorithms MOBIII and RPEA get the same number of best performances on test problem WFG1. Notice that the algorithm CMaPSO performs worse on the test problem WFG1 than the comparative algorithms NSGA-III, MOBIII, and RPEA.

Table 12 statistics the number of the best, better, similar, and worse performance of CMaPSO and the comparative algorithms NSGA-III, MOBIII, RPEA, and MyODEMR on the test suite WFG. The performance of all the adopted algorithms are evaluated by 45 test cases with 3, 5, 8, 10, and 15 objectives on the WFG test problem. The proposed algorithm has won 22 first-ranked test cases, accounting for 48.9% of the total.  Figure 7(b) shows the average performance score of all the comparative algorithms on the test suite WFG, where the proposed algorithm gets the best score. The algorithms NSGA-III, MOBIII, RPEA, and MyODEMR achieve 11, 2, 7, and 3 rank first test cases on the WFG test suite, accounting for 22%, 4.4%, 15.6%, and 6.7% of the total, respectively. The algorithm NSGA-III performs best in the comparative algorithms MOBII, RPEA, and MyODEMR. A total of 17 test cases are significantly better than the algorithm CMaPSO on the WFG test problem, but most of the test cases are inferior to the proposed algorithm. We can summarize the performance of the algorithm on the WFG test suite, which is obviously better than the employed comparative algorithm.

Table 13 summarizes the number of the best, better, similar, and worse performance of CMaPSO and the comparative algorithms NSGA-III, MOBIII, RPEA, and MyODEMR on the test suites DTLZ and WFG. From the table, the performance of the proposed algorithm CMaPSO ranks first on 38 test cases, accounting for almost half of the total.  Figure 6(d) illustrates the comprehensive average performance scores of all the comparative algorithms for different objective numbers on the test suites DTLZ and WFG, where the proposed algorithm achieves the best score on 5, 8, 10, and 15 objectives. And Figure 7(c) presents the average performance scores of all the comparative algorithms on the test suites DTLZ and WFG, and the proposed algorithm obtains the best score. The performance of algorithms NSGA-III, MOBIII, RPEA, and MyODEMR has achieved 20, 5, 12, and 5 test cases ranking first, accounting for 25%, 6.25%, 15%, and 6.25% of the total. The performance of NSGA-III is the best among the employed comparative algorithms. However, the performance algorithm NSGA-III on most test cases is inferior to the proposed algorithm CMaPSO. From all the abovementioned discussions, we can draw the conclusion that the performance of the proposed algorithm is better than the adopted comparative algorithms on the DTLZ and WFG test suits. Figures 5(f)–5(i) presents the approximate PF obtained by five many-objective evolutionary optimization algorithms on the 15 objectives of DTLZ1. Obviously, most of the comparative algorithms can converge to the true Pareto front, but the diversity of algorithm CMaPSO is better than the other algorithms.

### 4.6. Experiments of All Comparison Algorithms on the Test Set UF1-9

To sum up, we have verified the performance of all comparison algorithms on the many-objective test problems of the test suites DTLZ and WFG, as well as on the 3-objective test problem. Next, we use the complex test cases UF1-9 [44] to further prove the performance of the proposed algorithm on the 2 and 3 objective test problems. Tables 14 and 15 present the mean and standard deviation of the IGD values of the proposed algorithm and four recent multiobjective particle swarm optimization algorithms and four advanced many-objective evolutionary optimization algorithms on the test set UF1-9. The best value for each test case is highlighted with a bold background.

**Figure 8:**
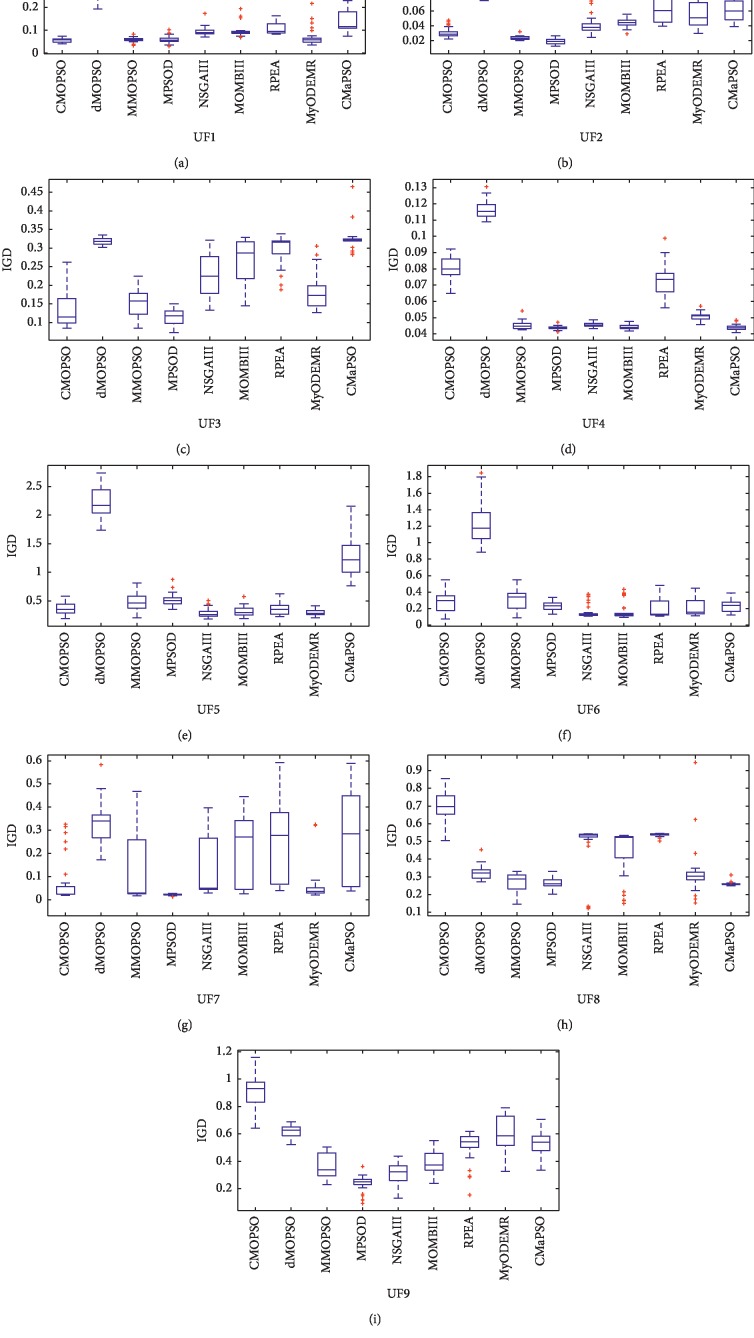
The performance of CMOPSO, dMOPSO, MMOPSO, MPSOD, NSGAIII, MOBIII, RPEA, MyODEMR, and CMaPSO on the test problem UF1-9 is presented by using the box plot.

In Table 14, the algorithm MPSOD obtained the best performance on the four test problems, and the proposed algorithm CMaPSO was the second, and the best performance was gained on the three test problems. CMOPSO and MMOPSO got one. Through comprehensive comparison and analysis, the performance of MPSOD is the best, MMOPSO and CMOPSO are better, and the comprehensive performance of CMOPSO is not as good as these three algorithms. From Table 15, the algorithms NSGAIII and MyODEMR achieved the best performance on three test problems, and the proposed algorithm CMaPSO obtained the best performance on two test problems. Although the algorithm MOMBIII got the best performance on one test question, its comprehensive performance was still excellent. Through the Wilcoxon rank sum test analysis, it is easy to find that NSGAIII performs best, MOMBIII performs better, and CMaPSO and MyODEMR perform similarly. In order to be able to observe the experimental results more visually, Figure 8 shows the experimental results of all comparison algorithms on test case UF1-9 by adopting the box graph. The box graph of these test problems can more vividly explain the performance of all comparison algorithms. It is easy to conclude that although the performance of the algorithm CMaPSO on the test problem UF1-9 is not the best among all the comparison algorithms, and it can effectively solve most complex multiobjective optimization problems.

### 4.7. Comparison of Running Time between Algorithms CMaPSO and CMOPSO

The algorithm CMaPSO and CMOPSO all employ the competition-based learning method to update the speed and position of the particles. In order to evaluate the performance of the proposed algorithm more comprehensively, it is necessary to compare the running time of the algorithm. From Table 16, it can be intuitively observed that the best values of all gray background are obtained by the algorithms CMaPSO. In this paper, CMaPSO can be regarded as the generalization of CMOPSO. The data in Table 16 shows that CMaPSO has shorter running time than CMOPSO on the multi/many-objective optimization problems. In addition, from Table 17, the significance test data of algorithm running time also shows that the proposed algorithm is more efficient than the algorithm CMOPSO.

## 5. Conclusion

In this paper, we have proposed a new multi/many-objective particle swarm optimization algorithms, termed as CMaPSO, which adopts competition-based learning to update the velocity and position of particles. The algorithm can be considered as the generalization of the algorithm CMOPSO. The main reasons are as follows: firstly, they all adopt the same learning method to update the speed and position of particles. Secondly, the algorithm CMOPSO does not perform well in solving many-objective optimization problems. Thirdly, the running time of the algorithm CMOPSO is relatively time-consuming. We mainly modify the environmental selection mechanism of CMOPSO and adopt the maximum and minimum angle between ordinary and extreme individuals to select excellent individuals to enter the next generation population. In order to tackle some complex problems more effectively, we use *θ*-dominance sorting and reference points regeneration strategy to further improve the algorithm.

To evaluate the competitiveness of the proposed algorithms, the proposed algorithm CMaPSO is compared with four recent multi/many-objective particle swarm optimization algorithms and compared with four many-objective evolutionary optimization algorithms, which have been empirically compared on eighty-nine standard benchmark test cases. The experimental results indicate that the algorithm CMaPSO has good robustness on most test problems and can effectively deal with multi/many-objective optimization problems. However, from the simulation comparison results, we found that the performance of the algorithm CMaPSO on the test case DTLZ7 is disappointing. In order to further improve the performance of the algorithm, we will continue to study the proposed algorithm CMaPSO and apply it to solve engineering problems in the future.

## Figures and Tables

**Algorithm 1:**
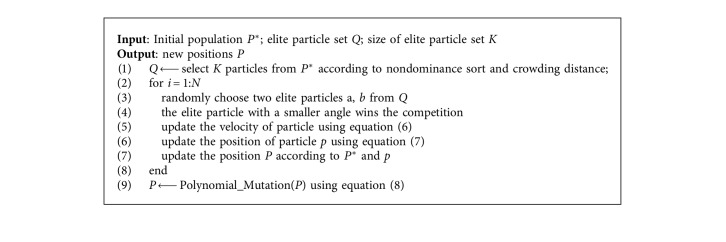
Competition-Based Learning.

**Algorithm 2:**
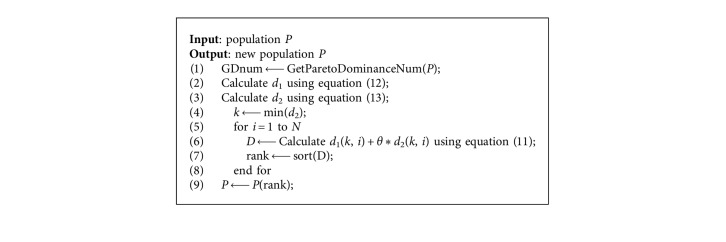
*θ*-dominance sorting.

**Algorithm 3:**
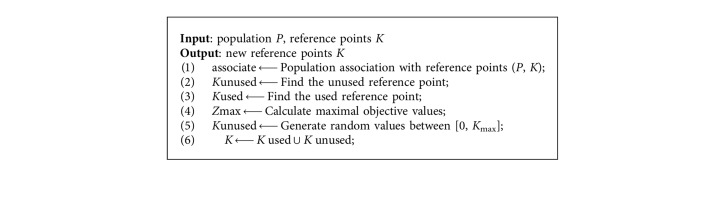
Reference point regeneration strategy.

**Algorithm 4:**
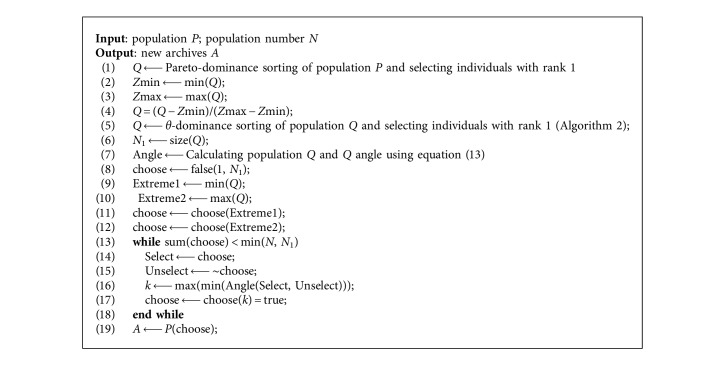
Archives updating.

**Algorithm 5:**
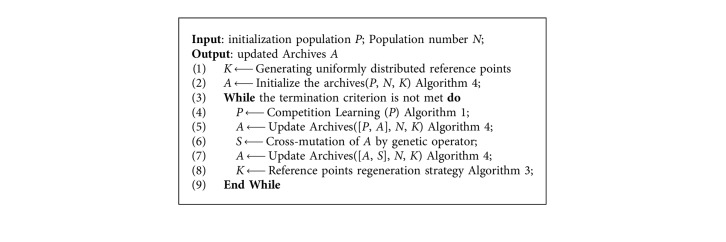
Framework of the proposed CMaPSO.

**Table 1:** The features of the test problems.

Problems	Features
DTLZ1	Linear, multimodal
DTLZ2	Concave
DTLZ3	Concave, multimodal
DTLZ4	Concave, biased
DTLZ5	Concave, degenerate
DTLZ6	Concave, degenerate, biased
DTLZ7	Mixed, disconnected, multimodal, scaled
WFG1	Mixed, biased, scaled
WFG2	Convex, disconnected, multimodal, nonseparable, scaled
WFG3	Linear, degenerate, nonseparable, scaled
WFG4	Concave, multimodal, scaled
WFG5	Concave, deceptive, scaled
WFG6	Concave, nonseparable, scaled
WFG7	Concave, biased, scaled
WFG8	Concave, biased, nonseparable, scaled
WFG9	Concave, biased, multimodal, deceptive, nonseparable, scaled
UF1	Complex Pareto set
UF2	Complex Pareto set
UF3	Complex Pareto set
UF4	Complex Pareto set, concave
UF5	Complex Pareto set, discrete
UF6	Complex Pareto set, discontinuous
UF7	Complex Pareto set
UF8	Complex Pareto set, concave
UF9	Complex Pareto set, discontinuous

**Table 2:** Test sets DTLZ and WFG decision variables settings.

Problems	*m*	*d*	Parameter
DTLZ1	3, 5, 8, 10, 15	*m* + *k* − 1	*k* = 5
DTLZ2- DTLZ6	3, 5, 8, 10, 15	*m* + *k* − 1	*k* = 10
DTLZ7	3, 5, 8, 10, 15	*m* + *k* − 1	*k* = 20
WFG	3, 5, 8, 10, 15	*k* + *l*	*k* = 2^∗^(m-1); 1=20
UF1-UF7	2	30	
UF8-UF9	2	30	

**Table 3:** Set different population sizes for different dimensions and a uniform number of evaluations.

Dimension number	Population number	Evaluation
3	100	150000
5	150	150000
8	200	150000
10	250	150000
15	300	150000

**Table 4:** The mean and standard deviation of the IGD value of the proposed algorithms and the four recently comparative algorithms CMOPSO, MMOPSO, MPSOD, and dMOPSO on DTLZ for 3, 5, 8, 10, and 15 objective problems, where the best value for each test case is highlighted with a bold background.

Problem	M	CMOPSO	dMOPSO	MMOPSO	MPSOD	CMaPSO
DTLZ1	3	3.2557*e* − 2(1.79*e* − 2)−	7.8004*e* − 1(6.93*e* − 1)−	5.4064*e* − 2(8.59*e* − 2)−	**1.9100e − 2(5.02e − 5)+**	2.2047*e* − 2(2.92*e* − 4)
	5	7.3425*e* + 0(3.95*e* + 0)−	7.6675*e* − 1(7.32*e* − 1)−	1.9539*e* + 0(1.99*e* + 0)−	6.7643*e* − 2(3.40*e* − 2)−	**6.4419e − 2(5.43e − 3)**
	8	3.9661*e* + 1(1.55*e* + 1)−	4.6021*e* − 1(1.83*e* − 1)−	1.1739*e* + 1(6.20*e* + 0)−	3.5917*e* − 1(7.11*e* − 2)−	**1.0696e − 1(5.96e − 3)**
	10	9.1496*e* + 1(2.26*e* + 1)−	1.2893*e* + 0(1.71*e* + 0)−	1.3584*e* + 1(3.95*e* + 0)−	7.6499*e* − 1(1.39*e* − 1)−	**1.3077e − 1(1.58e − 2)**
	15	1.0964*e* + 2 (1.26*e* + 1) −	9.2687*e* − 1(9.42*e* − 1)−	1.6867*e* + 1(7.47*e* + 0)−	1.2428*e* + 0(4.81*e* − 1)−	**1.3692e − 1(8.31e − 3)**

DTLZ2	3	5.7035*e* − 2(3.05*e* − 4)=	1.3362*e* − 1(7.19*e* − 3)−	6.9615*e* − 2(1.86*e* − 3)−	**5.4516e − 2(1.23e − 5)+**	6.0866*e* − 2(1.22*e* − 3)
	5	3.6973*e* − 1(3.88*e* − 2)−	2.9030*e* − 1(4.75*e* − 3)−	2.4978*e* − 1(1.01*e* − 2)−	1.9659*e* − 1(6.55*e* − 5)=	**1.9525e − 1(1.20e − 3)**
	8	2.3305*e* + 0(2.70*e* − 2)−	5.1260*e* − 1(3.01*e* − 2)−	7.3105*e* − 1(6.33*e* − 2)−	**3.1787e − 1(3.28e − 4)+**	3.5837*e* − 1(1.79*e* − 3)
	10	2.3737*e* + 0(1.60*e* − 2)−	6.0004*e* − 1(3.10*e* − 2)−	1.1142*e* + 0(9.31*e* − 2)−	4.4485*e* − 1(5.84*e* − 4)−	**4.2631e − 1(2.22e − 3)**
	15	2.4967*e* + 0(1.68*e* − 2)−	8.2668*e* − 1(3.60*e* − 2)−	1.4850*e* + 0(1.96*e* − 1)−	5.3230*e* − 1(7.13*e* − 4)−	**4.9904e − 1(1.88e − 3)**

DTLZ3	3	3.4943*e* + 1(1.33*e* + 1)−	3.7248*e* + 0(4.47*e* + 0)−	7.3776*e* − 2(8.27*e* − 3)−	7.6661*e* + 0(3.46*e* + 0)−	**6.2885e − 2(6.68e − 3)**
	5	1.4470*e* + 2(1.53*e* + 1)−	1.2027*e* + 2(5.87*e* + 1)−	5.0745*e* + 1(5.00*e* + 1)−	1.8499*e* + 1(3.69*e* + 0)−	**2.1712e − 1(1.90e − 2)**
	8	4.3773*e* + 2(1.09*e* + 2)−	1.6852*e* + 2(5.82*e* + 1)−	1.6750*e* + 2(3.70*e* + 1)−	3.3532*e* + 1(4.90*e* + 0)−	**6.2463e − 1(2.48e − 1)**
	10	5.3570*e* + 2(1.46*e* + 2)−	1.7975*e* + 2(1.55*e* + 1)−	1.9185*e* + 2(2.35*e* + 1)−	4.8642*e* + 1(5.54*e* + 0)−	**1.9282e + 0(1.17e + 0)**
	15	7.3422*e* + 2(6.36*e* + 0)−	1.8714*e* + 2(1.26*e* + 1)−	1.8323*e* + 2(4.26*e* + 1)−	6.1343*e* + 1(8.37*e* + 0)−	**1.1990e + 0(7.43e − 1)**

DTLZ4	3	6.2273*e* − 2(3.06*e* − 3)+	2.2990*e* − 1(4.42*e* − 2)+	7.0481*e* − 2(1.90*e* − 3)+	**5.4519e − 2(1.24e − 5)+**	3.3993*e* − 1(3.21*e* − 1)
	5	4.1558*e* − 1(5.73*e* − 2)−	4.4812*e* − 1(2.54*e* − 2)−	2.3859*e* − 1(9.52*e* − 3)−	1.9596*e* − 1(1.04*e* − 4)=	**1.9522e − 1(9.00e − 4)**
	8	1.5137*e* + 0(1.31*e* − 1)−	5.8493*e* − 1(1.32*e* − 2)−	7.8410*e* − 1(1.11*e* − 1)−	**3.4118e − 1(1.38e − 3)=**	3.5373*e* − 1(1.59*e* − 3)
	10	1.3490*e* + 0(8.33*e* − 2)−	6.5214*e* − 1(1.78*e* − 2)−	1.3628*e* + 0(1.18*e* − 1)−	4.5450*e* − 1(5.10*e* − 4)−	**4.2690e − 1(4.12e − 3)**
	15	1.5053*e* + 0(7.64*e* − 2)−	7.5203*e* − 1(8.43*e* − 3)−	1.9333*e* + 0(3.48*e* − 1)−	5.7137*e* − 1(2.72*e* − 3)−	**5.3276e − 1(4.13e − 3)**

DTLZ5	3	**5.6927e − 3(8.30e − 4)+**	4.0628*e* − 2(5.35*e* − 3)−	6.1386*e* − 3(4.10*e* − 4)=	3.2522*e* − 2(1.17*e* − 3)−	6.3719*e* − 3(4.23*e* − 4)
	5	3.1111*e* − 1(2.57*e* − 2)−	**2.9915e − 2(4.86e − 3)+**	7.1303*e* − 2(1.83*e* − 2)−	8.5261*e* − 2(3.41*e* − 3)−	3.6560*e* − 2(6.96*e* − 3)
	8	1.8356*e* + 0(4.73*e* − 1)−	**2.9288e − 2(5.87e − 3)+**	1.7165*e* − 1(5.24*e* − 2)−	1.2868*e* − 1(9.95*e* − 3)−	5.4626*e* − 2(9.29*e* − 3)
	10	1.7715*e* + 0(7.11*e* − 1)−	**2.3843e − 2(5.25e − 3)+**	1.2075*e* − 1(3.69*e* − 2)−	1.7173*e* − 1(1.78*e* − 2)−	4.8186*e* − 2(8.22*e* − 3)
	15	1.7061*e* + 0(2.95*e* − 1)−	**2.7307e − 2(5.83e − 3)+**	1.7347*e* − 1(9.17*e* − 2)−	1.9137*e* − 1(3.52*e* − 2)−	4.7407*e* − 2(4.20*e* − 3)

DTLZ6	3	**4.2165e − 3(4.46e − 5)+**	3.3835*e* − 2(1.32*e* − 5)−	6.9594*e* − 3(9.60*e* − 4)=	3.3198*e* − 2(3.59*e* − 4)−	6.8460*e* − 3(6.24*e* − 4)
	5	3.8651*e* + 0(1.57*e* + 0)−	**3.2017e − 2(6.18e − 6)+**	2.9603*e* − 1(2.36*e* − 1)−	8.6575*e* − 2(1.18*e* − 3)+	1.9242*e* − 1(4.37*e* − 2)
	8	9.6427*e* + 0(1.81*e* − 1)−	**2.4404e − 2(2.53e − 4)+**	4.0488*e* − 1(2.42*e* − 1)−	1.2025*e* − 1(4.51*e* − 4)+	2.5237*e* − 1(5.01*e* − 2)
	10	9.7001*e* + 0(2.54*e* − 1)−	**2.4908e − 2(5.63e − 4)+**	5.4698*e* − 1(4.21*e* − 1)−	1.1575*e* − 1(2.34*e* − 4)+	2.2817*e* − 1(1.31*e* − 1)
	15	9.7485*e* + 0(1.53*e* − 1)−	**5.2210e − 2(5.22e − 6)+**	6.6979*e* − 1(5.08*e* − 1)−	1.4965*e* − 1(3.22*e* − 2)+	2.2625*e* − 1(2.45*e* − 1)

DTLZ7	3	**6.4645e − 2(1.71e − 3)+**	1.4252*e* − 1(2.36*e* − 2)+	8.5361*e* − 2(5.39*e* − 3)+	1.3306*e* − 1(1.85*e* − 3)+	1.5317*e* − 1(8.45*e* − 2)
	5	5.2563*e* − 1(2.25*e* − 2)+	5.4125*e* − 1(6.41*e* − 2)+	**3.6133e − 1(8.47e − 3)+**	6.2844*e* − 1(9.43*e* − 3)+	2.0956*e* + 0(7.85*e* − 1)
	8	5.9164*e* + 0(2.86*e* + 0)−	1.3807*e* + 0(1.35*e* − 1)+	**8.2129e − 1(3.09e − 2)+**	1.7184*e* + 0(3.57*e* − 1)+	2.8717*e* + 0(1.14*e* + 0)
	10	1.6003*e* + 1(5.01*e* + 0)−	1.6656*e* + 0(1.67*e* − 1)+	**1.5255e + 0(1.27e − 1)+**	2.1238*e* + 0(6.55*e* − 1)+	9.5705*e* + 0(3.19*e* + 0)
	15	6.5560*e* + 1(6.40*e* + 0)−	2.9213*e* + 0(3.20*e* − 1)+	**2.4919e + 0(3.91e − 1)+**	2.8822*e* + 0(4.36*e* − 1)+	1.5214*e* + 1(3.32*e* + 0)

+/−/=		6/29/0	14/21/0	7/28/0	14/19/2	

**Table 5:** The mean and standard deviation of the IGD value of the proposed algorithm and the four recently comparative algorithms CMOPSO, MMOPSO, MPSOD, and dMOPSO on WFG for 3, 5, 8, 10, and 15 objective problems, where the best value for each test case is highlighted with a bold background.

Problem	M	CMOPSO	dMOPSO	MMOPSO	MPSOD	CMaPSO
WFG1	3	1.5022*e* + 0(8.42*e* − 3)−	1.5274*e* + 0(6.04*e* − 3)−	4.3581*e* − 1(4.58*e* − 2)−	1.4777*e* + 0(3.20*e* − 2)−	**3.6797e − 1(6.06e − 2)**
	5	2.0348*e* + 0(1.90*e* − 2)−	2.0337*e* + 0(1.12*e* − 2)−	1.5417*e* + 0(4.96*e* − 2)=	1.9401*e* + 0(2.34*e* − 2)−	**1.5343e + 0(1.87e − 1)**
	8	2.6729*e* + 0(5.37*e* − 2)−	2.7149*e* + 0(1.02*e* − 2)−	2.3994*e* + 0(4.93*e* − 2)−	2.6159*e* + 0(1.69*e* − 2)−	**1.9306e + 0(9.28e − 2)**
	10	3.0866*e* + 0(2.20*e* − 2)−	3.1181*e* + 0(2.13*e* − 2)−	2.8852*e* + 0(5.91*e* − 2)−	3.0500*e* + 0(2.39*e* − 2)−	**2.6358e + 0(1.22e − 1)**
	15	4.0310*e* + 0(3.21*e* − 2)−	4.2140*e* + 0(3.10*e* − 2)−	3.8640*e* + 0(4.63*e* − 2)−	3.9929*e* + 0(2.72*e* − 2)−	**3.7283e + 0(7.91e − 2)**

WFG2	3	1.8125*e* − 1(8.54*e* − 3)−	3.7120*e* − 1(1.97*e* − 2)−	2.3566*e* − 1(1.26*e* − 2)−	1.9821*e* − 1(4.74*e* − 3)−	**1.6900e − 1(2.59e − 3)**
	5	6.4846*e* − 1(2.79*e* − 2)−	6.9837*e* − 1(3.03*e* − 2)−	7.8138*e* − 1(4.76*e* − 2)−	6.3753*e* − 1(3.77*e* − 2)−	**4.6603e − 1(6.91e − 3)**
	8	1.2562*e* + 0(3.65*e* − 2)−	1.4097*e* + 0(5.78*e* − 2)−	1.4180*e* + 0(6.18*e* − 2)−	1.1999*e* + 0(3.65*e* − 2)−	**1.0521e + 0(4.00e − 2)**
	10	1.5717*e* + 0(5.30*e* − 2)−	1.6534*e* + 0(2.39*e* − 2)−	1.5513*e* + 0(3.78*e* − 2)−	1.4113*e* + 0(5.47*e* − 2)−	**1.1252e + 0(3.35e − 2)**
	15	2.4815*e* + 0(2.93*e* − 1)−	2.6033*e* + 0(6.95*e* − 2)−	2.0643*e* + 0(9.59*e* − 2)−	1.9621*e* + 0(4.47*e* − 2)−	**1.6533e + 0(7.00e − 2)**

WFG3	3	1.9304*e* − 1(1.56*e* − 2)−	5.0349*e* − 1(7.21*e* − 2)−	**1.0427e − 1(1.24e − 2)=**	2.7890*e* − 1(2.65*e* − 2)−	1.0706*e* − 1(7.68*e* − 3)
	5	1.0736*e* + 0(9.85*e* − 2)−	7.9881*e* − 1(5.77*e* − 2)−	5.7070*e* − 1(8.62*e* − 2)−	8.3271*e* − 1(4.10*e* − 2)−	**3.8881e − 1(7.07e − 2)**
	8	2.2141*e* + 0(1.14*e* − 1)−	2.3638*e* + 0(6.11*e* − 1)−	1.2723*e* + 0(1.52*e* − 1)−	1.4738*e* + 0(3.54*e* − 2)−	**5.1806e − 1(8.01e − 2)**
	10	2.8920*e* + 0(1.42*e* − 1)−	4.1413*e* + 0(2.61*e* − 1)−	1.5555*e* + 0(2.18*e* − 1)−	1.8289*e* + 0(5.14*e* − 2)−	**6.7278e − 1(9.57e − 2)**
	15	4.9914*e* + 0(6.64*e* − 1)−	8.9915*e* + 0(2.49*e* + 0)−	2.8363*e* + 0(7.44*e* − 1)−	2.3692*e* + 0(5.44*e* − 2)−	**9.6840e − 1(7.37e − 2)**

WFG4	3	2.7079*e* − 1(2.97*e* − 3)−	3.2880*e* − 1(1.19*e* − 2)−	2.9431*e* − 1(1.69*e* − 2)−	2.6878*e* − 1(3.91*e* − 3)−	**2.4216e − 1(3.15e − 3)**
	5	**1.0795e + 0(2.11e − 2)=**	1.5872*e* + 0(1.58*e* − 1)−	1.2103*e* + 0(3.12*e* − 2)−	1.4754*e* + 0(1.97*e* − 2)−	1.1111*e* + 0(1.17*e* − 2)
	8	**2.8804e + 0(2.52e − 2)=**	7.4798*e* + 0(3.60*e* − 1)−	3.2326*e* + 0(4.10*e* − 2)−	4.7715*e* + 0(9.97*e* − 2)−	3.0092*e* + 0(2.50*e* − 2)
	10	4.2454*e* + 0(6.57*e* − 2)=	1.0133*e* + 1(1.55*e* − 1)−	4.5236*e* + 0(3.79*e* − 2)−	6.5960*e* + 0(1.21*e* − 1)−	**4.2453e + 0(2.07e − 2)**
	15	7.9002*e* + 0(1.11*e* − 1)=	1.5922*e* + 1(5.08*e* − 1)−	8.2037*e* + 0(7.88*e* − 2)−	9.0792*e* + 0(3.48*e* − 1)−	**7.8890e + 0(3.35e − 1)**

WFG5	3	2.6458*e* − 1(8.21*e* − 3)−	3.1650*e* − 1(3.26*e* − 2)−	2.8914*e* − 1(1.08*e* − 2)−	2.5515*e* − 1(9.62*e* − 4)−	**2.3862e − 1(3.81e − 3)**
	5	**1.0560e + 0(9.16e − 3)+**	1.3479*e* + 0(3.99*e* − 2)−	1.2652*e* + 0(2.90*e* − 2)−	1.5195*e* + 0(7.72*e* − 3)−	1.1126*e* + 0(1.35*e* − 2)
	8	**2.9384e + 0(3.29e − 2)+**	4.6484*e* + 0(2.25*e* − 1)−	3.4768*e* + 0(7.43*e* − 2)−	4.0103*e* + 0(1.64*e* − 1)−	3.1077*e* + 0(2.64*e* − 2)
	10	**4.1166e + 0(3.84e − 2)=**	6.8581*e* + 0(1.97*e* − 1)−	4.7322*e* + 0(6.47*e* − 2)−	5.4337*e* + 0(1.23*e* − 1)−	4.2940*e* + 0(5.03*e* − 2)
	15	7.6413*e* + 0(1.14*e* − 1)−	1.0395*e* + 1(2.98*e* − 1)−	8.3772*e* + 0(1.16*e* − 1)−	8.1415*e* + 0(9.91*e* − 2)−	**7.1937e + 0(9.64e − 2)**

WFG6	3	2.5950*e* − 1(8.18*e* − 3)−	2.9186*e* − 1(1.76*e* − 2)−	3.2614*e* − 1(1.46*e* − 2)−	2.8432*e* − 1(1.11*e* − 2)−	**2.4022e − 1(4.65e − 3)**
	5	1.1636*e* + 0(3.08*e* − 2)=	2.7199*e* + 0(1.35*e* − 1)−	1.2643*e* + 0(1.98*e* − 2)−	1.4793*e* + 0(5.45*e* − 2)−	**1.1512e + 0(7.89e − 3)**
	8	3.1267*e* + 0(4.98*e* − 2)=	8.4369*e* + 0(4.82*e* − 1)−	3.4341*e* + 0(4.46*e* − 2)−	4.6085*e* + 0(3.11*e* − 1)−	**3.1219e + 0(3.29e − 2)**
	10	**4.3428e + 0(3.50e − 2)=**	1.0817*e* + 1(2.06*e* − 1)−	4.6880*e* + 0(4.17*e* − 2)−	6.3171*e* + 0(2.85*e* − 1)−	4.3853*e* + 0(5.38*e* − 2)
	15	7.7888*e* + 0(9.19*e* − 2)−	1.6862*e* + 1(2.16*e* − 1)−	8.2301*e* + 0(8.32*e* − 2)−	8.9450*e* + 0(2.52*e* − 1)−	**7.4678e + 0(1.54e − 1)**

WFG7	3	2.3377*e* − 1(3.70*e* − 3)=	4.1408*e* − 1(1.16*e* − 2)−	2.8324*e* − 1(1.06*e* − 2)−	2.6031*e* − 1(2.03*e* − 3)−	**2.3088e − 1(4.14e − 3)**
	5	1.1356*e* + 0(2.42*e* − 2)=	2.1718*e* + 0(1.67*e* − 1)−	1.2244*e* + 0(3.73*e* − 2)−	1.4645*e* + 0(1.92*e* − 2)−	**1.1329e + 0(1.50e − 2)**
	8	**2.9881e + 0(2.59e − 2)+**	8.0059*e* + 0(5.46*e* − 1)−	3.4421*e* + 0(4.91*e* − 2)−	4.4225*e* + 0(1.60*e* − 1)−	3.1161*e* + 0(2.14*e* − 2)
	10	**4.2298e + 0(2.95e − 2)+**	1.0927*e* + 1(2.66*e* − 1)−	4.6792*e* + 0(4.96*e* − 2)−	6.0794*e* + 0(8.89*e* − 2)−	4.3651*e* + 0(3.94*e* − 2)
	15	7.6036*e* + 0(8.04*e* − 2)−	1.6080*e* + 1(2.70*e* − 1)−	8.2605*e* + 0(7.27*e* − 2)−	8.1167*e* + 0(1.85*e* − 1)−	**7.2438e + 0(1.31e − 1)**

WFG8	3	3.0896*e* − 1(8.31*e* − 3)−	5.2524*e* − 1(1.86*e* − 2)−	3.4639*e* − 1(8.27*e* − 3)−	2.9225*e* − 1(5.07*e* − 3)−	**2.8088e − 1(9.10e − 3)**
	5	1.2601*e* + 0(2.36*e* − 2)−	1.7185*e* + 0(1.05*e* − 1)−	1.2889*e* + 0(2.74*e* − 2)−	1.3809*e* + 0(1.20*e* − 2)−	**1.1628e + 0(6.08e − 3)**
	8	3.3381*e* + 0(1.61*e* − 2)−	7.7007*e* + 0(3.06*e* − 1)−	3.6005*e* + 0(1.07*e* − 1)−	3.8116*e* + 0(4.78*e* − 2)−	**3.1612e + 0(2.83e − 2)**
	10	4.5975*e* + 0(8.74*e* − 2)=	1.0607*e* + 1(2.12*e* − 1)−	4.8154*e* + 0(9.00*e* − 2)−	5.4392*e* + 0(1.08*e* − 1)−	**4.4489e + 0(3.53e − 2)**
	15	8.1480*e* + 0(1.03*e* − 1)−	1.5893*e* + 1(3.03*e* − 1)−	8.4762*e* + 0(1.36*e* − 1)−	8.8058*e* + 0(2.00*e* − 1)−	**7.8886e + 0(2.45e − 1)**

WFG9	3	**2.2829e − 1(2.82e − 3)+**	2.8053*e* − 1(7.44*e* − 3)−	2.9606*e* − 1(1.42*e* − 2)−	2.6147*e* − 1(1.36*e* − 2)−	2.4682*e* − 1(9.13*e* − 3)
	5	**1.0789e + 0(1.58e − 2)=**	1.8778*e* + 0(1.26*e* − 1)−	1.3602*e* + 0(1.05*e* − 1)−	1.4301*e* + 0(2.11*e* − 2)−	1.1105*e* + 0(7.62*e* − 3)
	8	3.2033*e* + 0(5.01*e* − 2)−	6.7459*e* + 0(1.26*e* − 1)−	3.7661*e* + 0(8.50*e* − 2)−	3.9769*e* + 0(1.86*e* − 1)−	**3.0804e + 0(2.19e − 2)**
	10	4.4527*e* + 0(5.42*e* − 2)−	9.5509*e* + 0(2.31*e* − 1)−	5.0222*e* + 0(4.49*e* − 2)−	5.3584*e* + 0(1.73*e* − 1)−	**4.1480e + 0(4.35e − 2)**
	15	7.9157*e* + 0(4.31*e* − 2)−	1.3970*e* + 1(1.14*e* + 0)−	8.5996*e* + 0(1.19*e* − 1)−	8.4536*e* + 0(2.16*e* − 1)−	**7.4027e + 0(9.57e − 2)**

+/−/=		8/29/8	0/45/0	0/43/2	0/45/0	

**Table 6:** Significance test between the proposed algorithm CMaPSO and the comparative algorithms on the DTLZ test problems.

	CMOPSO	dMOPSO	MMOPSO	MPSOD	CMaPSO
Rank first	3	8	4	5	15
Better (+)	5	14	6	13	
Same (=)	1	0	2	3	
Worse (−)	29	21	27	19	

**Table 7:** Significance test between the proposed algorithm CMaPSO and other comparison algorithms on the WFG test problems.

	CMOPSO	dMOPSO	MMOPSO	MPSOD	CMaPSO
Rank first	10	0	1	0	34
Better (+)	5	0	0	0	
Same (=)	12	0	2	0	
Worse (−)	28	45	43	45	

**Table 8:** Significance test between the proposed algorithm CMaPSO and the comparison algorithms on all test problems.

	CMOPSO	dMOPSO	MMOPSO	MPSOD	CMaPSO
Rank first	13	8	5	5	49
Better (+)	10	14	6	13	
Same (=)	13	0	4	3	
Worse (−)	57	66	70	64	

**Table 9:** The mean and standard deviation of the IGD value of the four advanced many-objective evolutionary optimization algorithms on the DTLZ test problems. The best value for each test case is highlighted with a bold background.

Problem	M	NSGAIII	MOMBIII	RPEA	MyODEMR	CMaPSO
DTLZ1	3	**2.0565e − 2(1.04e − 5)+**	2.0619*e* − 2(8.18*e* − 5)+	1.6814*e* − 1(5.04*e* − 2)−	4.2157*e* − 2(3.41*e* − 2)−	2.2047*e* − 2(2.92*e* − 4)
	5	**6.3383e − 2(5.69e − 5)+**	6.6678*e* − 2(9.94*e* − 3)−	1.8383*e* − 1(3.37*e* − 2)−	8.7627*e* − 2(2.86*e* − 2)−	6.4419*e* − 2(5.43*e* − 3)
	8	1.1143*e* − 1(1.83*e* − 2)=	2.1921*e* − 1(2.54*e* − 2)−	2.4231*e* − 1(2.91*e* − 2)−	2.0962*e* − 1(2.52*e* − 2)−	**1.0696e − 1(5.96e − 3)**
	10	1.6571*e* − 1(5.13*e* − 2)−	2.1222*e* − 1(3.29*e* − 2)−	2.5486*e* − 1(3.20*e* − 2)−	3.3665*e* − 1(6.77*e* − 2)−	**1.3077e − 1(1.58e − 2)**
	15	1.8382*e* − 1(1.10*e* − 1)−	2.7085*e* − 1(1.26*e* − 2)−	2.6497*e* − 1(4.63*e* − 2)−	1.3754*e* − 1(2.15*e* − 2)=	**1.3692e − 1(8.31e − 3)**

DTLZ2	3	**5.4464e − 2(4.13e − 7)+**	5.4492*e* − 2(7.81*e* − 6)+	1.0882*e* − 1(5.73*e* − 3)−	6.9240*e* − 2(3.21*e* − 3)−	6.0866*e* − 2(1.22*e* − 3)
	5	1.9490*e* − 1(8.21*e* − 6)=	1.9522*e* − 1(2.60*e* − 4)=	2.8356*e* − 1(3.09*e* − 2)−	2.0436*e* − 1(2.42*e* − 3)−	**1.9415e − 1(1.20e − 3)**
	8	3.6070*e* − 1(9.57*e* − 2)=	**3.2542e − 1(7.00e − 4)+**	3.8254*e* − 1(1.06*e* − 2)−	3.5330*e* − 1(4.43*e* − 3)=	3.5837*e* − 1(1.79*e* − 3)
	10	5.0117*e* − 1(7.63*e* − 2)−	4.5504*e* − 1(9.49*e* − 4)−	4.4131*e* − 1(8.14*e* − 3)−	**4.2515e − 1(5.01e − 3)=**	4.2631*e* − 1(2.22*e* − 3)
	15	6.6601*e* − 1(7.66*e* − 2)−	7.9236*e* − 1(6.58*e* − 2)−	5.4126*e* − 1(2.56*e* − 3)−	5.6685*e* − 1(1.61*e* − 2)−	**4.9904e − 1(1.88e − 3)**

DTLZ3	3	**5.4576e − 2(1.14e − 4)+**	5.4600*e* − 2(3.55*e* − 5)+	2.0403*e* − 1(2.78*e* − 2)−	2.4784*e* − 1(2.11*e* − 1)−	6.2885*e* − 2(6.68*e* − 3)
	5	**1.9545e − 1(4.91e − 4)+**	1.9644*e* − 1(1.09*e* − 3)+	4.5246*e* − 1(4.01*e* − 2)−	6.4675*e* − 1(3.12*e* − 1)−	2.1712*e* − 1(1.90*e* − 2)
	8	6.6757*e* − 1(1.04*e* + 0)−	**4.1902e − 1(1.44e − 1)+**	6.8299*e* − 1(6.66*e* − 2)−	1.1522*e* + 0(6.07*e* − 2)−	6.2463*e* − 1(2.48*e* − 1)
	10	3.4566*e* + 0(2.42*e* + 0)−	**6.5314e − 1(1.20e − 1)+**	8.2396*e* − 1(9.59*e* − 2)+	1.2320*e* + 0(1.66*e* − 2)+	1.9282*e* + 0(1.17*e* + 0)
	15	4.5793*e* + 1(2.46*e* + 1)−	1.0840*e* + 0(2.34*e* − 2)+	**1.0639e + 0(2.51e − 1)+**	1.2840*e* + 0(1.44*e* − 2)−	1.1990*e* + 0(7.43*e* − 1)

DTLZ4	3	1.0318*e* − 1(1.54*e* − 1)+	1.0321*e* − 1(1.54*e* − 1)+	5.7672*e* − 1(3.08*e* − 1)−	**6.9716e − 2(1.65e − 3)+**	3.3993*e* − 1(3.21*e* − 1)
	5	2.4182*e* − 1(9.90*e* − 2)−	2.8891*e* − 1(1.14*e* − 1)−	3.8703*e* − 1(2.15*e* − 1)−	2.2383*e* − 1(5.72*e* − 3)−	**1.9522e − 1(9.00e − 4)**
	8	3.7941*e* − 1(1.06*e* − 1)−	3.6120*e* − 1(4.19*e* − 2)−	3.9606*e* − 1(3.51*e* − 2)−	3.7977*e* − 1(5.46*e* − 3)−	**3.5373e − 1(1.59e − 3)**
	10	4.7630*e* − 1(4.28*e* − 2)−	4.5749*e* − 1(3.48*e* − 4)−	4.4578*e* − 1(6.26*e* − 3)−	4.7513*e* − 1(1.10*e* − 2)−	**4.2690e − 1(4.12e − 3)**
	15	5.4388*e* − 1(4.87*e* − 2)−	6.0064*e* − 1(6.02*e* − 3)−	5.4767*e* − 1(3.31*e* − 3)−	6.2169*e* − 1(2.24*e* − 3)−	**5.3276e − 1(4.13e − 3)**

DTLZ5	3	1.3276*e* − 2(1.68*e* − 3)−	2.5140*e* − 2(3.41*e* − 5)−	2.0755*e* − 2(4.05*e* − 3)−	3.7309*e* − 2(9.09*e* − 3)−	**6.3719e − 3(4.23e − 4)**
	5	3.0252*e* − 1(2.37*e* − 1)−	2.7061*e* − 1(5.20*e* − 3)−	5.1907*e* − 2(9.00*e* − 3)−	8.8047*e* − 1(5.26*e* − 2)−	**3.6560e − 2(6.96e − 3)**
	8	3.7565*e* − 1(1.07*e* − 1)−	2.8819*e* − 1(1.66*e* − 1)−	8.3767*e* − 2(3.01*e* − 2)−	1.4984*e* + 0(5.73*e* − 2)−	**5.4626e − 2(9.29e − 3)**
	10	4.1017*e* − 1(7.95*e* − 2)−	6.7472*e* − 1(1.10*e* − 1)−	8.3944*e* − 2(1.93*e* − 2)−	1.5900*e* + 0(9.84*e* − 2)−	**4.8186e − 2(8.22e − 3)**
	15	9.6608*e* − 1(2.40*e* − 1)−	7.2006*e* − 1(2.90*e* − 2)−	1.1546*e* − 1(3.40*e* − 2)−	1.7978*e* + 0(3.24*e* − 1)−	**4.7407e − 2(4.20e − 3)**

DTLZ6	3	2.1027*e* − 2(1.92*e* − 3)−	2.5149*e* − 2(1.70*e* − 6)−	1.9479*e* − 2(3.42*e* − 3)−	6.3035*e* − 2(5.54*e* − 2)−	**6.8460e − 3(6.24e − 4)**
	5	3.3816*e* − 1(1.04*e* − 1)−	3.1434*e* − 1(1.33*e* − 5)−	**1.3371e − 1(3.60e − 2)+**	8.5385*e* − 1(9.81*e* − 2)−	1.9242*e* − 1(4.37*e* − 2)
	8	1.3884*e* + 0(1.17*e* + 0)−	5.1714*e* − 1(1.32*e* − 1)−	**1.7717e − 1(6.42e − 2)+**	1.1300*e* + 0(1.61*e* − 1)−	2.5237*e* − 1(5.01*e* − 2)
	10	2.9306*e* + 0(1.62*e* + 0)−	5.9585*e* − 1(1.14*e* − 1)−	2.2791*e* − 1(6.45*e* − 2)=	1.2125*e* + 0(1.43*e* − 1)−	**2.2717e − 1(1.31e − 1)**
	15	6.1288*e* + 0(1.05*e* + 0)−	6.7540*e* − 1(6.14*e* − 2)−	**1.9664e − 1(6.15e − 2)+**	1.4495*e* + 0(6.06*e* − 1)−	2.2625*e* − 1(2.45*e* − 1)

DTLZ7	3	**7.6242e − 2(3.24e − 3)+**	2.2087*e* − 1(2.31*e* − 1)−	2.9105*e* − 1(2.30*e* − 1)−	2.5739*e* − 1(3.68*e* − 2)−	1.5317*e* − 1(8.45*e* − 2)
	5	**3.3576e − 1(1.70e − 2)+**	4.4758*e* − 1(7.53*e* − 2)+	1.4291*e* + 0(1.63*e* − 1)+	8.7415*e* − 1(6.57*e* − 2)+	2.0956*e* + 0(7.85*e* − 1)
	8	**8.1348e − 1(4.32e − 2)+**	2.5140*e* + 0(7.66*e* − 1)+	2.3518*e* + 0(3.34*e* − 1)+	3.4193*e* + 0(5.21*e* − 1)−	2.8717*e* + 0(1.14*e* + 0)
	10	**1.2525e + 0(1.36e − 1)+**	4.7379*e* + 0(7.11*e* − 1)+	2.2184*e* + 0(2.87*e* − 1)+	5.3261*e* + 0(4.22*e* − 1)+	9.5705*e* + 0(3.19*e* + 0)
	15	3.5665*e* + 0(3.34*e* − 1)+	1.1085*e* + 1(9.34*e* − 2)+	**2.4343e + 0(8.72e − 1)+**	1.0521*e* + 1(3.99*e* − 1)+	1.5214*e* + 1(3.32*e* + 0)

+/−/=		11/21/3	13/21/1	9/25/1	5/27/3	

**Table 10:** The mean and standard deviation of the IGD value of the four advanced many-objective evolutionary optimization algorithms on the WFG test problems. The best value for each test case is highlighted with a bold background.

Problem	M	NSGAIII	MOMBIII	RPEA	MyODEMR	CMaPSO
WFG1	3	**1.4828e − 1(2.49e − 3)+**	1.6620*e* − 1(5.47*e* − 3)+	9.2744*e* − 1(1.52*e* − 1)−	1.3417*e* + 0(1.82*e* − 1)−	3.6797*e* − 1(6.06*e* − 2)
	5	6.4684*e* − 1(3.78*e* − 2)+	**5.8287e − 1(1.61e − 1)+**	1.0331*e* + 0(2.28*e* − 1)+	2.0874*e* + 0(2.46*e* − 1)−	1.5343*e* + 0(1.87*e* − 1)
	8	1.3976*e* + 0(7.24*e* − 2)+	**1.3016e + 0(1.42e − 1)+**	1.5068*e* + 0(1.96*e* − 1)+	3.3522*e* + 0(4.90*e* − 1)−	1.9306*e* + 0(9.28*e* − 2)
	10	2.0023*e* + 0(7.95*e* − 2)+	1.7141*e* + 0(1.15*e* − 1)+	**1.6465e + 0(4.47e − 2)+**	3.8284*e* + 0(5.35*e* − 1)−	2.6358*e* + 0(1.22*e* − 1)
	15	3.1092*e* + 0(1.19*e* − 1)+	2.3870*e* + 0(1.30*e* − 1)+	**2.3827e + 0(1.27e − 1)+**	4.6443*e* + 0(2.96*e* − 1)−	3.7283*e* + 0(7.91*e* − 2)

WFG2	3	**1.6470e − 1(6.72e − 4)+**	2.0194*e* − 1(6.16*e* − 3)−	3.6533*e* − 1(3.55*e* − 2)−	4.5895*e* − 1(1.21*e* − 1)−	1.6900*e* − 1(2.59*e* − 3)
	5	4.7149*e* − 1(1.75*e* − 3)−	5.7165*e* − 1(1.42*e* − 1)−	7.8005*e* − 1(1.13*e* − 1)−	8.2489*e* − 1(1.70*e* − 1)−	**4.6603e − 1(6.91e − 3)**
	8	1.2751*e* + 0(3.16*e* − 1)−	1.2626*e* + 0(1.40*e* − 1)−	1.2647*e* + 0(1.04*e* − 1)−	1.5215*e* + 0(2.79*e* − 1)−	**1.0521e + 0(4.00e − 2)**
	10	1.6707*e* + 0(1.94*e* − 1)−	2.0888*e* + 0(4.73*e* − 1)−	1.4623*e* + 0(1.79*e* − 1)−	1.5048*e* + 0(8.71*e* − 2)−	**1.1252e + 0(3.35e − 2)**
	15	2.1508*e* + 0(1.51*e* − 1)−	8.2290*e* + 0(1.17*e* + 0)−	2.2949*e* + 0(4.78*e* − 1)−	3.0521*e* + 0(7.59*e* − 1)−	**1.6533e + 0(7.00e − 2)**

WFG3	3	8.3210*e* − 2(1.98*e* − 2)+	8.7194*e* − 2(4.77*e* − 3)+	**3.8867e − 2(2.52e − 3)+**	4.4170*e* − 1(8.55*e* − 1)−	1.0706*e* − 1(7.68*e* − 3)
	5	4.1981*e* − 1(7.22*e* − 2)−	1.5880*e* + 0(1.01*e* − 1)−	**4.7314e − 2(3.24e − 3)+**	5.2690*e* + 0(7.44*e* − 2)−	3.8881*e* − 1(7.07*e* − 2)
	8	9.1391*e* − 1(2.26*e* − 1)−	8.0883*e* + 0(1.11*e* − 1)−	**6.2806e − 2(4.62e − 3)+**	8.3924*e* + 0(1.29*e* − 1)−	5.1806*e* − 1(8.01*e* − 2)
	10	2.6070*e* + 0(1.52*e* + 0)−	9.8077*e* + 0(1.12*e* − 1)−	**7.4803e − 2(1.78e − 2)+**	1.0188*e* + 1(3.34*e* − 1)−	6.7278*e* − 1(9.57*e* − 2)
	15	3.7180*e* + 0(1.79*e* + 0)−	1.5405*e* + 1(1.42*e* − 1)−	**2.5292e − 1(8.11e − 2)+**	1.5672*e* + 1(1.76*e* − 1)−	9.6840*e* − 1(7.37*e* − 2)

WFG4	3	**2.2082e − 1(3.05e − 5)+**	2.4583*e* − 1(8.40*e* − 3)=	4.2990*e* − 1(5.53*e* − 2)−	2.7243*e* − 1(1.20*e* − 2)−	2.4216*e* − 1(3.15*e* − 3)
	5	1.1738*e* + 0(5.34*e* − 4)−	1.4715*e* + 0(2.57*e* − 1)−	1.2874*e* + 0(3.45*e* − 2)−	1.1384*e* + 0(1.71*e* − 2)−	**1.1111e + 0(1.17e − 2)**
	8	3.1688*e* + 0(5.54*e* − 1)−	3.7620*e* + 0(2.54*e* − 1)−	3.0544*e* + 0(5.59*e* − 2)−	3.0160*e* + 0(4.24*e* − 2)=	**3.0092e + 0(2.50e − 2)**
	10	4.8174*e* + 0(1.69*e* − 1)−	7.1181*e* + 0(6.87*e* − 1)−	4.3969*e* + 0(7.21*e* − 2)−	4.4493*e* + 0(8.51*e* − 2)−	**4.2453e + 0(2.07e − 2)**
	15	8.8091*e* + 0(4.82*e* − 1)−	1.7970*e* + 1(2.11*e* + 0)−	7.8842*e* + 0(6.43*e* − 2)=	8.6756*e* + 0(1.15*e* − 1)−	**7.8810e + 0(3.35e − 1)**

WFG5	3	**2.2990e − 1(3.37e − 5)+**	2.4460*e* − 1(6.10*e* − 3)−	3.9181*e* − 1(3.73*e* − 2)−	2.9952*e* − 1(9.80*e* − 3)−	2.3862*e* − 1(3.81*e* − 3)
	5	1.1646*e* + 0(3.35*e* − 4)−	1.2602*e* + 0(2.99*e* − 2)−	1.2474*e* + 0(4.90*e* − 2)−	1.1418*e* + 0(1.20*e* − 2)=	**1.1126e + 0(1.35e − 2)**
	8	2.9408*e* + 0(2.41*e* − 3)+	3.5800*e* + 0(7.16*e* − 2)−	3.0665*e* + 0(3.83*e* − 2)+	**2.9863e + 0(2.87e − 2)+**	3.1077*e* + 0(2.64*e* − 2)
	10	4.7349*e* + 0(8.84*e* − 3)−	6.5255*e* + 0(2.19*e* − 1)−	4.4372*e* + 0(8.84*e* − 2)−	4.3173*e* + 0(3.59*e* − 2)=	**4.2940e + 0(5.03e − 2)**
	15	8.0003*e* + 0(5.40*e* − 2)−	2.5317*e* + 1(1.22*e* + 0)−	7.6767*e* + 0(7.70*e* − 2)−	8.0252*e* + 0(3.24*e* − 2)−	**7.1937e + 0(9.64e − 2)**

WFG6	3	**2.2516e − 1(1.33e − 3)+**	2.3983*e* − 1(6.51*e* − 3)=	4.7244*e* − 1(8.63*e* − 2)−	2.8904*e* − 1(3.28*e* − 2)−	2.4022*e* − 1(4.65*e* − 3)
	5	1.1664*e* + 0(8.31*e* − 4)=	1.4798*e* + 0(2.15*e* − 1)−	1.6724*e* + 0(2.56*e* − 1)−	1.1727*e* + 0(2.88*e* − 2)=	**1.1512e + 0(7.89e − 3)**
	8	2.9554*e* + 0(4.23*e* − 3)+	3.7423*e* + 0(1.69*e* − 2)−	3.4378*e* + 0(1.56*e* − 1)−	**2.9837e + 0(2.41e − 2)+**	3.1219*e* + 0(3.29*e* − 2)
	10	4.7823*e* + 0(1.14*e* − 2)−	7.1299*e* + 0(5.60*e* − 2)−	4.6581*e* + 0(9.25*e* − 2)−	4.4373*e* + 0(5.43*e* − 2)=	**4.3853e + 0(5.38e − 2)**
	15	1.1085*e* + 1(5.47*e* − 1)−	1.6092*e* + 1(2.29*e* + 0)−	7.8701*e* + 0(9.14*e* − 2)−	8.1375*e* + 0(1.86*e* − 1)−	**7.4678e + 0(1.54e − 1)**

WFG7	3	**2.2106e − 1(6.94e − 5)+**	2.3205*e* − 1(1.00*e* − 2)=	5.3389*e* − 1(1.02*e* − 1)−	2.8956*e* − 1(1.13*e* − 2)−	2.3088*e* − 1(4.14*e* − 3)
	5	1.1751*e* + 0(4.29*e* − 4)−	1.3428*e* + 0(2.36*e* − 1)−	1.6856*e* + 0(1.46*e* − 1)−	1.1859*e* + 0(1.59*e* − 2)−	**1.1329e + 0(1.50e − 2)**
	8	**2.9697e + 0(6.98e − 3)+**	3.7402*e* + 0(1.59*e* − 2)−	3.5080*e* + 0(1.56*e* − 1)−	3.0879*e* + 0(4.12*e* − 2)=	3.1161*e* + 0(2.14*e* − 2)
	10	4.8138*e* + 0(3.06*e* − 2)−	6.7608*e* + 0(3.78*e* − 1)−	4.6440*e* + 0(1.67*e* − 1)−	4.5280*e* + 0(5.27*e* − 2)−	**4.3651e + 0(3.94e − 2)**
	15	1.0458*e* + 1(7.63*e* − 1)−	1.4015*e* + 1(2.08*e* + 0)−	7.9997*e* + 0(8.23*e* − 2)−	8.3747*e* + 0(3.39*e* − 2)−	**7.2438e + 0(1.31e − 1)**

WFG8	3	**2.3431e − 1(1.40e − 3)+**	2.8959*e* − 1(2.51*e* − 3)−	4.6968*e* − 1(4.60*e* − 2)−	2.9584*e* − 1(5.32*e* − 3)−	2.8088*e* − 1(9.10*e* − 3)
	5	**1.1461e + 0(6.16e − 4)=**	2.9829*e* + 0(2.30*e* − 2)−	1.5205*e* + 0(6.39*e* − 2)−	1.1519*e* + 0(1.79*e* − 2)=	1.1628*e* + 0(6.08*e* − 3)
	8	3.4504*e* + 0(5.59*e* − 1)−	3.8210*e* + 0(1.16*e* − 1)−	3.4439*e* + 0(1.78*e* − 1)−	**3.0529e + 0(3.78e − 2)+**	3.1612*e* + 0(2.83*e* − 2)
	10	5.0070*e* + 0(3.78*e* − 1)−	7.2435*e* + 0(8.51*e* − 2)−	4.8263*e* + 0(1.69*e* − 1)−	5.4932*e* + 0(2.10*e* − 1)−	**4.4489e + 0(3.53e − 2)**
	15	9.3500*e* + 0(5.01*e* − 1)−	1.9697*e* + 1(2.05*e* + 0)−	8.0531*e* + 0(1.17*e* − 1)−	1.0313*e* + 1(9.71*e* − 2)−	**7.8886e + 0(2.45e − 1)**

WFG9	3	**2.3336e − 1(1.44e − 2)+**	2.7000*e* − 1(1.50*e* − 2)−	3.9451*e* − 1(5.41*e* − 2)−	2.9471*e* − 1(7.23*e* − 3)−	2.4682*e* − 1(9.13*e* − 3)
	5	1.1330*e* + 0(5.26*e* − 3)=	2.6498*e* + 0(1.75*e* − 1)−	1.2971*e* + 0(1.14*e* − 1)−	1.1141*e* + 0(2.39*e* − 2)=	**1.1105e + 0(7.62e − 3)**
	8	**2.9408e + 0(1.80e − 2)+**	3.7030*e* + 0(3.45*e* − 2)−	3.5023*e* + 0(2.83*e* − 1)−	2.9853*e* + 0(3.12*e* − 2)+	3.0804*e* + 0(2.19*e* − 2)
	10	4.5640*e* + 0(1.61*e* − 2)−	7.0156*e* + 0(1.15*e* − 1)−	4.9314*e* + 0(4.81*e* − 1)−	4.3104*e* + 0(3.19*e* − 2)−	**4.1480e + 0(4.35e − 2)**
	15	7.9258*e* + 0(1.66*e* − 1)−	2.6021*e* + 1(8.11*e* − 1)−	8.3650*e* + 0(2.09*e* − 1)−	8.1817*e* + 0(6.41*e* − 2)−	**7.4027e + 0(9.57e − 2)**

+/−/=		17/25/3	6/36/3	10/34/1	4/33/8	

**Table 11:** Significance test between CMaPSO and other comparison algorithms on the DTLZ test problems.

	NSGAIII	MOMBIII	RPEA	MyODEMR	CMaPSO
Rank first	9	3	5	2	16
Better (+)	11	13	9	5	
Same (≈)	3	1	1	3	
Worse (−)	21	21	25	27	

**Table 12:** Significance test between CMaPSO and other comparison algorithms on the WFG test problems.

	NSGAIII	MOMBIII	RPEA	MyODEMR	CMaPSO
Rank first	11	2	7	3	22
Better (+)	17	6	10	4	
Same (≈)	3	3	1	8	
Worse (−)	25	36	34	33	

**Table 13:** Significance test between CMaPSO and the comparison algorithms on the all test problems.

	NSGAIII	MOMBIII	RPEA	MyODEMR	CMaPSO
Rank first	20	5	12	5	38
Better (+)	28	19	19	9	
Same (≈)	6	4	2	11	
Worse (−)	46	57	59	60	

**Table 14:** The mean and standard deviation of the IGD value of the proposed algorithms and the four recently comparative algorithms CMOPSO, MMOPSO, MPSOD, and dMOPSO on UF1-9, where the best value for each test case is highlighted with a bold background.

Problem	M	CMOPSO	dMOPSO	MMOPSO	MPSOD	CMaPSO
UF1	2	5.7138*e* − 2 (1.83*e* − 2)+	3.7540*e* − 1 (7.68*e* − 2)−	5.9788*e* − 2 (1.53*e* − 2)+	**5.1749e − 2 (1.34e − 2)+**	1.3331*e* − 1 (3.73*e* − 2)
UF2	2	2.9899*e* − 2 (1.04*e* − 2)+	9.2730*e* − 2 (1.52*e* − 2)−	2.4096*e* − 2 (2.37*e* − 3)+	**1.8998e − 2 (2.46e − 3)+**	6.3018*e* − 2 (1.76*e* − 2)
UF3	2	1.2699*e* − 1 (3.38*e* − 2)+	3.1958*e* − 1 (5.65*e* − 3)=	**1.2233e − 1 (4.01e − 2)+**	1.2517*e* − 1 (1.90*e* − 2)+	3.1369*e* − 1 (2.23*e* − 2)
UF4	2	8.1806*e* − 2 (8.02*e* − 3)−	1.1693*e* − 1 (5.52*e* − 3)−	4.5258*e* − 2 (2.98*e* − 3)−	4.4709*e* − 2 (1.32*e* − 3)=	**4.4248e − 2 (2.67e − 3)**
UF5	2	**3.8937e − 1 (1.25e − 1)+**	2.1659*e* + 0 (2.83*e* − 1)−	5.1412*e* − 1 (2.03*e* − 1)+	5.3157*e* − 1 (1.15*e* − 1)+	1.1908*e* + 0 (2.65*e* − 1)
UF6	2	2.2563*e* − 1 (1.06*e* − 1)=	1.3225*e*+0 (3.58*e* − 1)−	3.4226*e* − 1 (1.48*e* − 1)−	2.2916*e* − 1 (5.69*e* − 2)=	**2.2525e − 1 (1.68e − 1)**
UF7	2	7.6681*e* − 2 (1.27*e* − 1)+	3.0330*e* − 1 (7.88*e* − 2)−	1.3358*e* − 1 (1.18*e* − 1)+	**1.9950e − 2 (3.37e − 3)+**	2.3489*e* − 1 (1.84*e* − 1)
UF8	3	6.8855*e* − 1 (9.86*e* − 2)−	3.2944*e* − 1 (3.23*e* − 2)−	2.4841*e* − 1 (7.90*e* − 2)−	2.2823*e* − 1 (6.93*e* − 2)=	**2.2780e − 1 (5.92e − 3)**
UF9	3	9.4259*e* − 1 (1.61*e* − 1)−	6.2794*e* − 1 (4.36*e* − 2)−	3.4257*e* − 1 (6.32*e* − 2)+	**2.4705e − 1 (5.94e − 2)+**	5.0482*e* − 1 (6.16*e* − 2)

+/−/=		5/3/1	0/7/2	6/3/0	6/0/3		

**Table 15:** The mean and standard deviation of the IGD value of the four advanced many-objective evolutionary optimization algorithms on the UF1-9 test problems. The best value for each test case is highlighted with a bold background.

Problem	M	NSGAIII	MOMBIII	RPEA	MyODEMR	CMaPSO
UF1	2	1.3356*e − *1 (2.19*e − *2)=	9.4162*e − *2 (1.62*e − *2)+	1.3086*e − *1 (3.66*e − *2)=	**6.2656e − 2 (3.88e − 2)+**	1.3331*e − *1 (3.73*e − *2)
UF2	2	**3.7800e − 2 (7.34e − 3)+**	4.7754*e − *2 (1.52*e − *2)+	6.5567*e − *2 (2.89*e − *2)−	6.1227*e − *2 (4.67*e − *2)=	6.3018*e − *2 (1.76*e − *2)
UF3	2	2.1073*e − *1 (4.71*e − *2)+	3.1511*e − *1 (6.13*e − *2)=	2.9777*e − *1 (3.22*e − *2)=	**1.9565e − 1 (6.36e − 2)+**	3.1369*e − *1 (2.23*e − *2)
UF4	2	4.6032*e − *2 (1.33*e − *3)−	4.6501*e − *2 (2.61*e − *3)−	7.5297*e − *2 (1.17*e − *2)−	5.1650*e − *2 (2.55*e − *3)−	**4.4248e − 2 (2.67e − 3)**
UF5	2	**2.7662e − 1 (7.32e − 2)+**	2.9237*e − *1 (1.00*e − *1)+	3.2235*e − *1 (1.15*e − *1)+	3.1381*e − *1 (6.56*e − *2)+	1.1908*e* + 0 (2.65*e − *1)
UF6	2	1.7038*e − *1 (1.09*e − *1)+	**1.7013e − 1 (9.07e − 2)+**	2.0888*e − *1 (1.11*e − *1)+	2.4345*e − *1 (1.21*e − *1)−	2.2525*e − *1 (1.68*e − *1)
UF7	2	1.3985*e − *1 (1.35*e − *1)+	2.2897*e − *1 (1.76*e − *1)=	2.4553*e − *1 (1.39*e − *1)−	**5.2280e − 2 (6.40e − 2)+**	2.3489*e − *1 (1.84*e − *1)
UF8	3	4.9871*e − *1 (1.03*e − *1)−	4.3170*e − *1 (1.26*e − *1)−	5.2668*e − *1 (4.79*e − *2)−	2.9866*e − *1 (4.61*e − *2)−	**2.2780e − 1 (5.92e − 3)**
UF9	3	**3.0157e − 1 (8.61e − 2)+**	3.6046*e − *1 (1.10*e − *1)+	5.0350*e − *1 (9.58*e − *2)=	5.8142*e − *1 (1.31*e − *1)−	5.0482*e − *1 (6.16*e − *2)

+/−/=		6/2/1	5/2/2	2/4/3	4/4/1		

**Table 16:** The running time of algorithm CMaPSO and algorithm CMOPSO is on the test suites DTLZ and WFG test suites, and the best value is highlighted in bold background.

Problem	M	CMOPSO	CMaPSO
DTLZ1	3	2.0325*e + *1(6.40*e + *0)−	**1.3247e + 1(7.85e − 1)**
	5	9.8638*e + *1(4.97*e − *1)−	**1.3337e + 1(1.19e + 0)**
	8	1.7133*e + *2(8.88*e + *1)−	**1.5771e + 1(9.33e − 1)**
	10	9.6345*e + *2(8.72*e + *1)−	**2.0261e + 1(1.96e + 0)**
	15	1.0586*e + *3(3.81*e + *1)−	**1.9567e + 1(1.75e + 0)**

DTLZ2	3	1.0213*e + *2(1.16*e + *1)−	**1.3558e + 1(9.22e − 1)**
	5	1.1368*e + *2(7.83*e + *0)−	**1.3941e + 1(1.33e + 0)**
	8	7.2410*e + *2(3.37*e + *1)−	**1.5809e + 1(1.15e + 0)**
	10	1.3434*e + *3(1.17*e + *2)−	**2.4512e + 1(2.05e + 0)**
	15	1.1525*e + *3(4.03*e + *1)−	**2.2854e + 1(2.69e + 0)**

DTLZ3	3	**1.3356e + 1(3.38e + 0)=**	1.4022*e + *1(9.61*e − *1)
	5	**1.2529e + 1(2.35e + 0)=**	1.3185*e + *1(5.12*e − *1)
	8	4.0956*e + *2(1.88*e + *1)−	**1.4730e + 1(8.98e − 1)**
	10	1.0847*e + *3(7.70*e + *1)−	**2.0134e + 1(1.48e + 0)**
	15	1.0725*e + *3(2.48*e + *1)−	**1.9188e + 1(2.33e + 0)**

DTLZ4	3	8.7389*e + *1(8.57*e + *0)−	**2.1440e + 1(1.72e + 0)**
	5	1.8360*e + *2(1.82*e + *1)−	**1.2812e + 1(1.03e + 0)**
	8	6.9939*e + *2(3.26*e + *1)−	**1.6309e + 1(1.06e + 0)**
	10	1.3385*e + *3(7.43*e + *1)−	**2.4079e + 1(1.69e + 0)**
	15	1.0910*e + *3(6.83*e + *1)−	**2.2716e + 1(2.43e + 0)**

DTLZ5	3	5.5077*e + *1(2.27*e + *0)−	**2.0834e + 1(1.82e + 0)**
	5	9.6019*e + *1(9.49*e + *0)−	**1.5241e + 1(2.38e + 0)**
	8	6.7374*e + *2(4.92*e + *1)−	**1.5641e + 1(1.02e + 0)**
	10	1.2522*e + *3(5.87*e + *1)−	**2.2997e + 1(8.48e − 1)**
	15	1.0647*e + *3(6.33*e + *1)−	**2.0185e + 1(2.56e + 0)**

DTLZ6	3	7.4785*e + *1(3.08*e + *0)−	**2.1424e + 1(1.94e + 0)**
	5	1.7705*e + *2(6.23*e + *1)−	**1.2330e + 1(9.39e − 1)**
	8	7.4953*e + *2(3.44*e + *1)−	**1.5401e + 1(9.24e − 1)**
	10	1.3524*e + *3(1.51*e + *2)−	**2.0092e + 1(1.94e + 0)**
	15	1.1064*e + *3(4.43*e + *1)−	**1.9240e + 1(2.52e + 0)**

DTLZ7	3	4.4794*e + *1(6.82*e + *0)−	**2.4811e + 1(2.15e + 0)**
	5	1.5473*e + *2(1.08*e + *1)−	**1.7914e + 1(1.61e + 0)**
	8	5.5592*e + *2(3.54*e + *1)−	**2.4724e + 1(3.07e + 0)**
	10	1.0898*e + *3(7.96*e + *1)−	**1.9551e + 1(2.98e + 0)**
	15	1.1207*e + *3(1.96*e + *1)−	**2.8312e + 1(1.30e + 1)**

WFG1	3	7.5295*e + *1(5.78*e + *0)−	**2.0274e + 1(2.07e + 0)**
	5	1.6252*e + *2(1.93*e + *1)−	**1.5829e + 1(2.06e + 0)**
	8	2.6396*e + *2(2.69*e + *1)−	**1.7167e + 1(1.95e + 0)**
	10	4.0521*e + *2(8.53*e + *1)−	**1.8244e + 1(3.50e + 0)**
	15	5.6740*e + *2(1.73*e + *1)−	**1.8881e + 1(1.99e + 0)**

WFG2	3	3.8167*e + *1(2.22*e + *0)−	**1.3256e + 1(1.46e + 0)**
	5	2.3355*e + *2(1.66*e + *1)−	**1.4563e + 1(1.38e + 0)**
	8	5.7765*e + *2(1.90*e + *1)−	**1.7866e + 1(1.28e + 0)**
	10	1.2167*e + *3(3.60*e + *2)−	**2.2871e + 1(2.67e + 0)**
	15	9.5575*e + *2(2.00*e + *1)−	**2.2693e + 1(1.62e + 0)**

WFG3	3	4.2085*e + *1(1.82*e + *0)−	**1.2114e + 1(9.71e − 1)**
	5	2.7558*e + *2(4.94*e + *0)−	**1.3580e + 1(1.13e + 0)**
	8	7.6980*e + *2(2.19*e + *1)−	**2.1984e + 1(1.91e + 0)**
	10	1.4993*e + *3(2.84*e + *2)−	**3.5400e + 1(1.10e + 1)**
	15	1.1370*e + *3(2.93*e + *1)−	**2.6364e + 1(3.04e + 0)**

WFG4	3	1.0979*e + *2(6.46*e + *0)−	**1.4193e + 1(1.04e + 0)**
	5	3.4257*e + *2(1.10*e + *1)−	**1.4548e + 1(1.12e + 0)**
	8	8.9442*e + *2(5.22*e + *1)−	**2.2418e + 1(1.48e + 0)**
	10	1.4845*e + *3(1.63*e + *2)−	**2.7789e + 1(3.35e + 0)**
	15	1.1123*e + *3(5.59*e + *1)−	**2.7387e + 1(4.26e + 0)**

WFG5	3	1.3188*e + *2(6.75*e + *0)−	**1.4279e + 1(2.29e + 0)**
	5	3.7246*e + *2(2.76*e + *1)−	**1.4887e + 1(2.55e + 0)**
	8	9.2150*e + *2(1.14*e + *2)−	**1.8684e + 1(1.16e + 0)**
	10	1.4429*e + *3(1.25*e + *2)−	**2.9439e + 1(4.44e + 0)**
	15	1.0793*e + *3(4.72*e + *1)−	**2.4731e + 1(3.08e + 0)**

WFG6	3	9.3599*e + *1(4.71*e + *0)−	**1.3490e + 1(1.75e + 0)**
	5	2.9378*e + *2(1.98*e + *1)−	**1.4815e + 1(1.63e + 0)**
	8	7.7411*e + *2(3.71*e + *1)−	**1.8815e + 1(1.32e + 0)**
	10	1.4829*e + *3(1.28*e + *2)−	**3.0236e + 1(4.03e + 0)**
	15	1.0765*e + *3(5.49*e + *1)−	**2.5458e + 1(2.88e + 0)**

WFG7	3	1.1370*e + *2(1.30*e + *1)−	**1.3340e + 1(8.87e − 1)**
	5	3.2237*e + *2(2.06*e + *1)−	**1.5460e + 1(1.01e + 0)**
	8	8.9250*e + *2(1.36*e + *2)−	**1.6997e + 1(1.76e + 0)**
	10	1.3971*e + *3(2.05*e + *2)−	**2.9195e + 1(6.42e + 0)**
	15	1.1366*e + *3(1.01*e + *2)−	**2.5141e + 1(2.82e + 0)**

WFG8	3	8.7390*e + *1(8.17*e + *0)−	**1.4061e + 1(2.43e − 1)**
	5	3.0043*e + *2(1.38*e + *1)−	**1.4979e + 1(1.15e + 0)**
	8	7.1609*e + *2(3.23*e + *1)−	**1.7472e + 1(1.58e + 0)**
	10	1.3283*e + *3(1.52*e + *2)−	**2.6407e + 1(2.45e + 0)**
	15	1.1042*e + *3(1.92*e + *1)−	**2.6856e + 1(2.65e + 0)**

WFG9	3	1.0228*e + *2(7.44*e + *0)−	**1.4616e + 1(2.02e − 1)**
	5	2.8967*e + *2(1.54*e + *1)−	**1.5815e + 1(1.41e + 0)**
	8	8.5906*e + *2(3.19*e + *2)−	**1.6439e + 1(1.29e + 0)**
	10	1.2128*e + *3(5.55*e + *1)−	**2.7584e + 1(2.53e + 0)**
	15	1.2783*e + *3(2.52*e + *2)−	**2.5133e + 1(2.04e + 0)**

**Table 17:** Significance test between CMaPSO and algorithm CMOPSO on the DTLZ and WFG test suites.

	CMOPSO	CMaPSO
Rank first	0	78
Better (+)	0	
Same (≈)	2	
Worse (−)	78	

## Data Availability

The data used to support the findings of this study are included within the article.

## Supplementary Materials

Supplementary Materials The mathematical expressions of the test sets DTLZ, WFG, and UF are presented in supplementary materials.
