# Supplementary material for: Multi/Many-Objective Particle Swarm Optimization Algorithm Based on Competition Mechanism
Source: Comput Intell Neurosci. 2020 Feb 19;2020:5132803. doi: 10.1155/2020/5132803 (PMC7063896; doi:10.1155/2020/5132803)
Supplement: Supplementary Materials — The mathematical expressions of the test sets DTLZ, WFG, and UF are presented in supplementary materials. [file 5132803.f1.docx]

**Supplementary Materials**

**DTLZ1-DTZL7**

**Function definitions**

• *D*: number of decision variables

• *M*: number of objectives

• **x** = *(*$x_{1}$*,*$x_{2}$*, …,*$x_{D}$*)*: decision vector

•$f_{i}$: *i*th objective function

**DTLZ1**

$$\mathrm{Min}\left\{ \begin{aligned} &f_{1}(x)=\frac{1}{2}x_{1}x_{2}\ldots x_{M-1}\left( 1+g\left( x_{M} \right) \right), \\ &f_{2}(x)=\frac{1}{2}x_{1}x_{2}\ldots\left( 1-x_{M-1} \right)\left( 1+g\left( x_{M} \right) \right), \\ & \vdots\\ &f_{M-1}\left( x \right)=\frac{1}{2}x_{1}\left( 1-x_{2} \right)\left( 1+g\left( x_{M} \right) \right), \\ &f_{M}\left( x \right)=\frac{1}{2}\left( 1-x_{1} \right)\left( 1+g\left( x_{M} \right) \right), \end{aligned} \right.$$

subject to $0\leq x_{i}\leq1$, for $i=1,2,\cdots,n$, with

$$g\left( x_{M} \right)=100\left[ \left| x_{M} \right|+\sum_{{x_{i}\in x}_{M}} {(x_{i}-0.5)}^{2}-\cos(20\pi(x_{i}-0.5)) \right].$$

where the number of decision variables is $D=M+k-1$, $k = 5$ is suggested here.

**DTLZ2**

$$\mathrm{Min}\left\{ \begin{aligned} &f_{1}(x)=\left( 1+g\left( x_{M} \right) \right)\cos(x_{1}\pi/{2)\cos(x_{2}\pi/{2)}}\ldots\cos(x_{M-2}\pi/{2)\cos(x_{M-1}\pi/{2)}}, \\ &f_{2}(x)=\left( 1+g\left( x_{M} \right) \right)\cos(x_{1}\pi/{2)\cos(x_{2}\pi/{2)}}\ldots\cos(x_{M-2}\pi/{2)\sin(x_{M-1}\pi/{2)}}, \\ & \vdots\\ &f_{M-1}\left( x \right)=\left( 1+g\left( x_{M} \right) \right)\cos(x_{1}\pi/{2)\sin{(x}_{2}\pi/{2)}}, \\ &f_{M}\left( x \right)=\left( 1+g\left( x_{M} \right) \right)\sin{(x}_{1}\pi/{2)}, \end{aligned} \right.$$

subject to $0\leq x_{i}\leq1$, for $i=1,2,\cdots,n$, with

$$g\left( x_{M} \right)=\sum_{{x_{i}\in x}_{M}} {(x_{i}-0.5)}^{2}.$$

where the number of decision variables is $D=M+k-1$, $k = 10$ is suggested here.

**DTLZ3**

$$\mathrm{Min}\left\{ \begin{aligned} &f_{1}(x)=\left( 1+g\left( x_{M} \right) \right)\cos(x_{1}\pi/{2)\cos(x_{2}\pi/{2)}}\ldots\cos(x_{M-2}\pi/{2)\cos(x_{M-1}\pi/{2)}}, \\ &f_{2}(x)=\left( 1+g\left( x_{M} \right) \right)\cos(x_{1}\pi/{2)\cos(x_{2}\pi/{2)}}\ldots\cos(x_{M-2}\pi/{2)\sin(x_{M-1}\pi/{2)}}, \\ & \vdots\\ &f_{M-1}\left( x \right)=\left( 1+g\left( x_{M} \right) \right)\cos(x_{1}\pi/{2)\sin{(x}_{2}\pi/{2)}}, \\ &f_{M}\left( x \right)=\left( 1+g\left( x_{M} \right) \right)\sin{(x}_{1}\pi/{2)}, \end{aligned} \right.$$

subject to $0\leq x_{i}\leq1$, for $i=1,2,\cdots,n$, with

$$g\left( x_{M} \right)=100\left[ \left| x_{M} \right|+\sum_{{x_{i}\in x}_{M}} {(x_{i}-0.5)}^{2}-\cos(20\pi(x_{i}-0.5)) \right].$$

where the number of decision variables is $D=M+k-1$, $k = 10$ is suggested here.

**DTLZ4**

$$\mathrm{Min}\left\{ \begin{aligned} &f_{1}(x)=\left( 1+g\left( x_{M} \right) \right)\cos(x_{1}^{\alpha}\pi/{2)\cos(x_{2}^{\alpha}\pi/{2)}}\ldots\cos(x_{M-2}^{\alpha}\pi/{2)\cos(x_{M-1}^{\alpha}\pi/{2)}}, \\ &f_{2}(x)=\left( 1+g\left( x_{M} \right) \right)\cos(x_{1}^{\alpha}\pi/{2)\cos(x_{2}^{\alpha}\pi/{2)}}\ldots\cos(x_{M-2}^{\alpha}\pi/{2)\sin(x_{M-1}^{\alpha}\pi/{2)}}, \\ & \vdots\\ &f_{M-1}\left( x \right)=\left( 1+g\left( x_{M} \right) \right)\cos(x_{1}^{\alpha}\pi/{2)\sin{(x}_{2}^{\alpha}\pi/{2)}}, \\ &f_{M}\left( x \right)=\left( 1+g\left( x_{M} \right) \right)\sin(x_{1}^{\alpha}\pi/{2)}, \end{aligned} \right.$$

subject to $0\leq x_{i}\leq1$, for $i=1,2,\cdots,n$, with

$$g\left( x_{M} \right)=\sum_{{x_{i}\in x}_{M}} {(x_{i}-0.5)}^{2}.$$

where the number of decision variables is $D=M+k-1$, $k = 10$ is suggested here.

**DTLZ5**

$$\mathrm{Min}\left\{ \begin{aligned} &f_{1}(x)=\left( 1+g\left( x_{M} \right) \right)\cos(\theta_{1}\pi/{2)\cos(\theta_{2}\pi/{2)}}\ldots\cos(\theta_{M-2}\pi/{2)\cos(\theta_{M-1}\pi/{2)}}, \\ &f_{2}(x)=\left( 1+g\left( x_{M} \right) \right)\cos(\theta_{1}\pi/{2)\cos(\theta_{2}\pi/{2)}}\ldots\cos(\theta_{M-2}\pi/{2)\sin(\theta_{M-1}\pi/{2)}}, \\ & \vdots\\ &f_{M-1}\left( x \right)=\left( 1+g\left( x_{M} \right) \right)\cos(\theta_{1}\pi/{2)\sin{(\theta}_{2}\pi/{2)}}, \\ &f_{M}\left( x \right)=\left( 1+g\left( x_{M} \right) \right)\sin{(\theta}_{1}\pi/{2)}, \end{aligned} \right.$$

subject to $0\leq x_{i}\leq1$, for $i=1,2,\cdots,n$, with

$\theta_{i}=\frac{\pi}{4\left( 1+g\left( r \right) \right)}\left( 1+2g\left( r \right)x_{i} \right),$for $i=2,3,\cdots,(M-1)$,

$$g\left( x_{M} \right)=\sum_{{x_{i}\in x}_{M}} {(x_{i}-0.5)}^{2}.$$

where the number of decision variables is $D=M+k-1$, $k = 10$ is suggested here.

**DTLZ6**

$$\mathrm{Min}\left\{ \begin{aligned} &f_{1}(x)=\left( 1+g\left( x_{M} \right) \right)\cos(\theta_{1}\pi/{2)\cos(\theta_{2}\pi/{2)}}\ldots\cos(\theta_{M-2}\pi/{2)\cos(\theta_{M-1}\pi/{2)}}, \\ &f_{2}(x)=\left( 1+g\left( x_{M} \right) \right)\cos(\theta_{1}\pi/{2)\cos(\theta_{2}\pi/{2)}}\ldots\cos(\theta_{M-2}\pi/{2)\sin(\theta_{M-1}\pi/{2)}}, \\ & \vdots\\ &f_{M-1}\left( x \right)=\left( 1+g\left( x_{M} \right) \right)\cos(\theta_{1}\pi/{2)\sin{(\theta}_{2}\pi/{2)}}, \\ &f_{M}\left( x \right)=\left( 1+g\left( x_{M} \right) \right)\sin{(\theta}_{1}\pi/{2)}, \end{aligned} \right.$$

subject to $0\leq x_{i}\leq1$, for $i=1,2,\cdots,n$, with

$\theta_{i}=\frac{\pi}{4\left( 1+g\left( r \right) \right)}\left( 1+2g\left( r \right)x_{i} \right),$for $i=2,3,\cdots,(M-1)$,

$$g\left( x_{M} \right)=\sum_{{x_{i}\in x}_{M}} {(x_{i})}^{0.1}.$$

where the number of decision variables is $D=M+k-1$, $k = 10$ is suggested here.

**DTLZ7**

$$\mathrm{Min}\left\{ \begin{aligned} &f_{1}(x)=x_{1}, \\ & \vdots\\ &f_{M-1}\left( x \right)=x_{M-1}, \\ &f_{M}\left( x \right)=\left( 1+g\left( x_{M} \right) \right)h(f_{1},f_{2},\cdots,f_{M-1},g), \end{aligned} \right.$$

subject to $0\leq x_{i}\leq1$, for $i=1,2,\cdots,n$, with

$h\left( f_{1},f_{2},\cdots,f_{M-1},g \right)=M-\sum_{i=1}^{M-1} \left[ \frac{f_{i}}{1+g}\left( 1+\sin\left( 3\pi f_{i} \right) \right) \right],$

$$g\left( x_{M} \right)=1+\frac{9}{\left| x_{M} \right|}\sum_{{x_{i}\in x}_{M}} x_{i}.$$

where the number of decision variables is $D=M+k-1$, $k = 20$ is suggested here.

**WFG1-WFG9**

| Problem | Type | Setting |
| --- | --- | --- |
| All | Constants | $S_{m=1:M}=2m$,$D=1$,$A_{1}=1$,$A_{2:M-1}=\left\{ \begin{aligned} 0, for WFG3 \\ 1, otherwise \end{aligned} \right.$ |
|  |  | The settings for $S_{1:M}$ ensures the Pareto optimal fronts have dissimilar tradeoff magnitudes, and the settings for $A_{1:M-1}$ ensures the Pareto optimal fronts are not degenerate, except in the case of WFG3, which has a one dimensional Pareto optimal front. |
| All | Domains | $z_{i=1:n},max=2i$ |
|  |  | The working parameters have domains of dissimilar magnitude. |
| WFG1 | Shape | $h_{m=1:M-1}={convex}_{m}$; $h_{M}={mixed}_{M}$(with $\alpha=1 and A=5$) |
|  | $t^{1}$ | $t_{i=1:k}^{1}=y_{i}$, $t_{i=k+1:n}^{1}=s\_linear$($y_{i},0.35$) |
|  | $t^{2}$ | $t_{i=1:k}^{2}=y_{i}$,$t_{i=k+1:n}^{2}=b\_flat(y_{i},0.8,0.75,0.85$) |
|  | $t^{3}$ | $t_{i=1:n}^{3}=b\_poly(y_{i},0.02)$ |
|  | $t^{4}$ | $t_{i=1:M-1}^{4}=r\_sum(\left\{ y_{\left( i-1 \right)k/(M-1)+1},\cdots,y_{ik/(M-1)} \right\}, \left\{ 2(\left( i-1 \right)k/(M-1)+1 ,\cdots,2ik/(M-1) \right\})$  $t_{M}^{4}=r\_sum(\left\{ y_{k+1},\cdots,y_{n} \right\},\left\{ 2\left( k+1 \right),\cdots,2n \right\})$ |
| WFG2 | Shape | $h_{m=1:M-1}={convex}_{m}$; $h_{M}={disc}_{M}$(with $\alpha=\beta=1 and A=5$) |
|  | $t^{1}$ | As $t^{1}$ from WFG1. (Linear shift.) |
|  | $t^{2}$ | $t_{i=1:k}^{2}=y_{i}$, $t_{i=k+1:k+l/2}^{2}=r\_nonsep(\left\{ y_{k+2\left( i-k \right)-1,} y_{k+2\left( i-k \right)} \right\},2$) |
|  | $t^{3}$ | $t_{i=1:M-1}^{3}=r\_sum(\left\{ y_{\left( i-1 \right)k/(M-1)+1},\cdots,y_{ik/(M-1)} \right\}, \left\{ 1 ,\cdots,1) \right\})$  $t_{M}^{3}=r\_sum(\left\{ y_{k+1},\cdots,y_{k+l/2} \right\},\left\{ 1,\cdots,1 \right\})$ |
| WFG3 | Shape | $h_{m=1:M}={linear}_{m}(degenerate)$; |
|  | $t^{1:3}$ | As $t^{1:3}$ from WFG2. (Linear shift, non-separable reduction, and weighted sum reduction.) |
| WFG4 | Shape | $h_{m=1:M}={concave}_{m}$; |
|  | $t^{1}$ | $t_{i=1:n}^{1}=s\_multi$($y_{i},30,10,0.35$) |
|  | $t^{2}$ | $t_{i=1:M-1}^{2}=r\_sum(\left\{ y_{\left( i-1 \right)k/(M-1)+1},\cdots,y_{ik/(M-1)} \right\}, \left\{ 1 ,\cdots,1 \right\})$  $t_{M}^{2}=r\_sum(\left\{ y_{k+1},\cdots,y_{n} \right\},\left\{ 1,\cdots,1 \right\})$ |
| WFG5 | Shape | $h_{m=1:M}={concave}_{m}$ |
|  | $t^{1}$ | $t_{i=1:n}^{1}=s\_decept$($y_{i},0.35,0.001,0.05$) |
|  | $t^{2}$ | As $t^{2}$ from WFG4. (Weighted sum reduction.) |
| WFG6 | Shape | $h_{m=1:M}={concave}_{m}$ |
|  | $t^{1}$ | As $t^{1}$ from WFG1. (Linear shift.) |
|  | $t^{2}$ | $t_{i=1:M-1}^{2}=r\_nonsep(\left\{ y_{\left( i-1 \right)k/(M-1)+1},\cdots,y_{ik/(M-1)} \right\}, k/{(M-1)})$  $t_{M}^{2}=r\_nonsep(\left\{ y_{k+1},\cdots,y_{n} \right\},l)$ |
| WFG7 | Shape | $h_{m=1:M}={concave}_{m}$ |
|  | $t^{1}$ | $t_{i=1:k}^{1}=b\_param(y_{i},r\_sum(\left\{ y_{k+1},\cdots,y_{n} \right\},\left\{ 1,\cdots,1 \right\}),\frac{0.98}{49.98},0.02,50)$  $t_{i=k+1:n}^{1}=y_{i}$ |
|  | $t^{2}$ | As $t^{1}$ from WFG1. (Linear shift.) |
|  | $t^{3}$ | As $t^{2}$ from WFG4. (Weighted sum reduction.) |
| WFG8 | Shape | $h_{m=1:M}={concave}_{m}$ |
|  | $t^{1}$ | $t_{i=1:k}^{1}=y_{i}$  $t_{i=k+1:n}^{1}=b\_param(y_{i},r\_sum(\left\{ y_{1},\cdots,y_{i-1} \right\},\left\{ 1,\cdots,1 \right\}),\frac{0.98}{49.98},0.02,50)$ |
|  | $t^{2}$ | As $t^{1}$ from WFG1. (Linear shift.) |
|  | $t^{3}$ | As $t^{2}$ from WFG4. (Weighted sum reduction.) |
| WFG9 | Shape | $h_{m=1:M}={concave}_{m}$ |
|  | $t^{1}$ | $t_{i=1:n-1}^{1}=b\_param(y_{i},r\_sum(\left\{ y_{i+1},\cdots,y_{n} \right\},\left\{ 1,\cdots,1 \right\}),\frac{0.98}{49.98},0.02,50)$  $t_{n}^{1}=y_{n}$ |
|  | $t^{2}$ | $t_{i=1:k}^{2}=s\_decept$($y_{i},0.35,0.001,0.05$)  $t_{i=k+1:n}^{2}=s\_multi$($y_{i},30,95,0.35$) |
|  | $t^{3}$ | As $t^{2}$ from WFG6. (Non-separable reduction.) |

The definition of test set WFG1-WFG9 comes from literature [41]. The shape functions and conversion functions involved in WFG1-WFG9 are shown in the following tables.

**Shape Functions**

| **Linear**  $\mathrm{linear}_{1}\left( x_{1},\cdots,x_{M-1} \right) = \prod_{i=1}^{M-1} x_{i}$  $\mathrm{linear}_{m=2:M-1}\left( x_{1},\cdots,x_{M-1} \right) =(\prod_{i=1}^{M-m} x_{i})(1-x_{M-m+1})$  $\mathrm{linear}_{M}\left( x_{1},\cdots,x_{M-1} \right) =1-x_{1}$  When $h_{m=1:M}=\mathrm{linear}_{m}$, the Pareto optimal front is a linear hyperplane, where $\sum_{m=1}^{M} h_{m}=1$. |
| --- |
| **Convex**  $\mathrm{Convex}_{1}\left( x_{1},\cdots,x_{M-1} \right) = \prod_{i=1}^{M-1} \left( 1-\cos\left( x_{i}\pi/2 \right) \right)$  $\mathrm{Convex}_{m=2:M-1}\left( x_{1},\cdots,x_{M-1} \right) =\left( \prod_{i=1}^{M-m} \left( 1-\cos\left( x_{i}\pi/2 \right) \right) \right)(1-\sin{(x}_{M-M+1}\pi/2))$  $\mathrm{Convex}_{M}\left( x_{1},\cdots,x_{M-1} \right) =1-\sin(x_{1}\pi/2)$  When $h_{m=1:M}=\mathrm{convex}_{m}$, the Pareto optimal front is purely convex. |
| **Concave**  $\mathrm{Concave}_{1}\left( x_{1},\cdots,x_{M-1} \right) = \prod_{i=1}^{M-1} \sin(x_{i}\pi/2)$  $\mathrm{Concave}_{m=2:M-1}\left( x_{1},\cdots,x_{M-1} \right) =\left( \prod_{i=1}^{M-m} \sin(x_{i}\pi/2) \right)\cos{(x}_{M-M+1}\pi/2)$  $\mathrm{Concave}_{M}\left( x_{1},\cdots,x_{M-1} \right) =\cos(x_{1}\pi/2)$  When $h_{m=1:M}=\mathrm{concave}_{m}$, the Pareto optimal front is purely concave, and a region of the hyper-sphere of radius one centred at the origin, where $\sum_{m=1}^{M} h_{m}^{2}=1$. |
| **Mixed convex/concave** $(\alpha>0,A\in\left\{ 1,2,\cdots\right\})$  $\mathrm{Mixed}_{M}\left( x_{1},\cdots,x_{M-1} \right) =\left( 1-x_{1}-\frac{\cos(2A\pi x_{1}+\pi/2)}{2A\pi} \right)^{\alpha}$  Causes the Pareto optimal front to contain both convex and concave segments, the number of which is controlled by A. The overall shape is controlled by $\alpha$: when$\alpha>1$ or when$\alpha<1$, the overall shape is convex or concave respectively. When $\alpha=1$, the overall shape is linear. |
| **Disconnected**$(\alpha,\beta>0,A\in\left\{ 1,2,\cdots\right\})$  ${disc}_{M}\left( x_{1},\cdots,x_{M-1} \right) =1-\left( x_{1} \right)^{\alpha}{cos}^{2}(A{(x_{1})}^{\beta}\pi)$  Causes the Pareto optimal front to have disconnected regions, the number of which is controlled by A. The overall shape is controlled by$\alpha$ (when$\alpha>1$ or when$\alpha<1$, the overall shape is concave or convex respectively, and when$\alpha=1$, the overall shape is linear), and $\beta$ influences the location of the disconnected regions (larger values push the location of disconnected regions towards larger values of $x_{1}$, and vice versa). |

**Transformation Functions**

| **Bias: Polynomial**$(\alpha>0,\alpha\neq1)$  $b\_poly\left( y,\alpha\right) =y^{\alpha}$  When$\alpha>1$ or when$\alpha<1$, $y$ is biased towards zero or towards one respectively. |
| --- |
| **Bias: Flat Region**$(A,B,C\in[0,1]$, $B<C$, $B=0$ $\Rightarrow$ $A=0\bigwedge C\neq1$, $C=1\Rightarrow A=1\bigwedge B\neq0)$  $b\_flat\left( y,A,B,C \right)=A+min(0,\left\lfloor y-B \right\rfloor)\frac{A\left( B-y \right)}{B}-min(0,\left\lfloor C-y \right\rfloor)\frac{(1-A)(y-C)}{1-C}$  Values of y between B and C (the area of the flat region) are all mapped to the value A. |
| **Bias: Parameter Dependent**($A\in\left( 0,1 \right), 0<B<C$)  $b\_param\left( y,y^{'},A,B,C \right) = y^{B+(C-B)v(u(y^{'}))}$  $v\left( u\left( y^{'} \right) \right)=A-(1-2u(y^{'}))\left\vert\left\lfloor0.5-u\left( y^{'} \right) \right\rfloor+A \right\vert$  A, B, C and the secondary parameter vector $y^{'}$ together determine the degree to which y is biased by being raised to an associated power: values of $u\left( y^{'} \right)\in[0,0.5]$ are mapped linearly onto $[B, B+(C-B)A]$, and values of $u\left( y^{'} \right)\in[0,0.5]$ are mapped linearly onto $[B+\left( C-B \right)A,C]$. |
| **Shift: Linear** ($A\in\left( 0,1 \right))$  $s\_linear\left( y,A \right)= \frac{\left\vert y-A \right\vert}{\left\vert\left\lfloor A-y \right\rfloor+A \right\vert}$  A is the value for which y is mapped to zero. |
| **Shift: Deceptive** ($A\in\left( 0,1 \right), 0<B\ll1, 0<C\ll1, A-B>0, A+B<1$)  $s\_linear\left( y,A,B,C \right)= 1+(\left\vert y-A \right\vert-B)\times\left( \frac{\left\lfloor y-A+B \right\rfloor(1-C+\frac{A-B}{B})}{A-B}+\frac{\left\lfloor A+B-y \right\rfloor(1-C+\frac{1-A-B}{B})}{1-A-B}+\frac{1}{B} \right)$  A is the value at which y is mapped to zero, and the global minimum of the transformation. B is the “aperture” size of the well/basin leading to the global minimum at A, and C is the value of the deceptive minima (there are always two deceptive minima). |
| **Shift: Multi-model** ($A\in\left\{ 1,2,\cdots\right\}, B\geq0,( 4A+2)\pi\geq4B, C\in\left( 0,1 \right)$)  $s\_multi\left( y,A,B,C \right)= \left( \frac{1+\cos\left[ ( 4A+2)\pi\left( 0.5-\frac{\left\vert y-C \right\vert}{2(\left\lfloor C-y \right\rfloor+C)} \right) \right]+4B\left( \frac{\left\vert y-C \right\vert}{2(\left\lfloor C-y \right\rfloor+C)} \right)^{2}}{B+2} \right)$  A controls the number of minima, B controls the magnitude of the “hill sizes” of the multi-modality, and C is the value for which y is mapped to zero. When $B=0$, $2A+1$ values of y (one at C) are mapped to zero, and when $B\neq0$, there are $2A$ local minima, and one global minimum at C. Larger values of A and smaller values of B create more difficult problems. |
| **Reduction: Weighted Sum** ($\left\vert w \right\vert=\left\vert y \right\vert,\omega_{1},\cdots,\omega_{\left\vert y \right\vert}>0)$  $r\_sum\left( y,w \right)=(\sum_{i=1}^{\left\vert y \right\vert} w_{i}y_{i})/\sum_{i=1}^{\left\vert y \right\vert} w_{i}$  By varying the constants of the weight vector **w**, EAs can be forced to treat parameters differently. |
| **Reduction: Non-separable** ($A\in\left\{ 1,\cdots,\left\vert y \right\vert\right\},\left\vert y \right\vert mod A=0$)  $r\_nonsep(y,A)=\frac{\sum_{j=1}^{\left\vert y \right\vert} (y_{j}+\sum_{k=0}^{A-2} \left\vert y_{j}-y_{1+\left( j+k \right) mod \left\vert y \right\vert} \right\vert)}{\frac{\left\vert y \right\vert}{A}\left\lceil A/2 \right\rceil(1+2A-2\left\lceil A/2 \right\rceil)}$  A controls the degree of non-separability (noting that $r\_nonsep\left( y,1 \right)=r\_sum\left( y,\left\{ 1,\cdots,1 \right\} \right)$). |

**UF1-UF9**

• *D*: number of decision variables

• *M*: number of objectives

**UF1**

$$min\left\{ \begin{aligned} &f_{1}=x_{1}+\frac{2}{\left| J_{1} \right|}\sum_{j\in J_{1}} \left[ x_{j}-\sin(6\pi x_{1}+\frac{j\pi}{n}) \right]^{2} \\ &f_{2}=1-\sqrt{x_{1}}+\frac{2}{\left| J_{2} \right|}\sum_{j\in J_{2}} \left[ x_{j}-\sin(6\pi x_{1}+\frac{j\pi}{n}) \right]^{2} \end{aligned} \right.$$

where $J_{1}=\{j|j is odd and 2\leq j\leq n\}$ and $J_{2}=\{j|j is even and 2\leq j\leq n\}$, the number of decision variables is $D=30$, and the number of objectives is $M = 2$. The search space is $\left[ 0,1 \right]\times\left[ -1,1 \right]^{n-1}$.

**UF2**

$$min\left\{ \begin{aligned} &f_{1}=x_{1}+\frac{2}{\left| J_{1} \right|}\sum_{j\in J_{1}} y_{j}^{2} \\ &f_{2}=1-\sqrt{x_{1}}+\frac{2}{\left| J_{2} \right|}\sum_{j\in J_{2}} y_{j}^{2} \end{aligned} \right.$$

where

$$y_{j}=\left\{ \begin{aligned} &x_{j}-\left[ 0.3x_{1}^{2}\cos\left( 24\pi x_{1}+\frac{4j\pi}{n} \right)+0.6x_{1} \right]\cos\left( 6\pi x_{1}+\frac{j\pi}{n} \right) j\in J_{1} \\ x_{j}-\left[ 0.3x_{1}^{2}\cos\left( 24\pi x_{1}+\frac{4j\pi}{n} \right)+0.6x_{1} \right]\sin\left( 6\pi x_{1}+\frac{j\pi}{n} \right) j\in J_{2} \end{aligned} \right.$$

$J_{1}=\{j|j is odd and 2\leq j\leq n\}$ and $J_{2}=\{j|j is even and 2\leq j\leq n\}$, and the number of decision variables is $D=30$, and the number of objectives is $M = 2$. The search space is $\left[ 0,1 \right]\times\left[ -1,1 \right]^{n-1}$.

**UF3**

$$min\left\{ \begin{aligned} &f_{1}=x_{1}+\frac{2}{\left| J_{1} \right|}\left( 4\sum_{j\in J_{1}} y_{j}^{2}-2\prod_{j\in J_{1}} \cos\left( \frac{20y_{j}\pi}{\sqrt{j}} \right)+2 \right) \\ &f_{2}=1-\sqrt{x_{1}}+\frac{2}{\left| J_{2} \right|}\left( 4\sum_{j\in J_{2}} y_{j}^{2}-2\prod_{j\in J_{2}} \cos\left( \frac{20y_{j}\pi}{\sqrt{j}} \right)+2 \right) \end{aligned} \right.$$

where

$$y_{j}=x_{1}^{0.5\left( 1+\frac{3(j-2)}{n-2} \right)}, j=2,\cdots,n,$$

$J_{1}=\{j|j is odd and 2\leq j\leq n\}$ and $J_{2}=\{j|j is even and 2\leq j\leq n\}$, and the number of decision variables is $D=30$, and the number of objectives is $M = 2$. The search space is $\left[ 0,1 \right]^{n}$.

**UF4**

$$min\left\{ \begin{aligned} &f_{1}=x_{1}+\frac{2}{\left| J_{1} \right|}\sum_{j\in J_{1}} h(y_{j}) \\ &f_{2}=1-x_{1}^{2}+\frac{2}{\left| J_{2} \right|}\sum_{j\in J_{2}} h(y_{j}) \end{aligned} \right.$$

where

$$y_{j}=x_{j}-\sin\left( 6\pi x_{1}+\frac{j\pi}{n} \right), j=2,\cdots,n,$$

$J_{1}=\{j|j is odd and 2\leq j\leq n\}$ and $J_{2}=\{j|j is even and 2\leq j\leq n\}$, and the number of decision variables is $D=30$, and the number of objectives is $M = 2$. The search space is $\left[ 0,1 \right]\times\left[ -2,2 \right]^{n-1}$.

**UF5**

$$min\left\{ \begin{aligned} &f_{1}=x_{1}+\left( \frac{1}{2N}+\varepsilon\right)\left| \sin\left( 2N\pi x_{1} \right) \right|+\frac{2}{\left| J_{1} \right|}\sum_{j\in J_{1}} h(y_{j}) \\ &f_{2}=1-x_{1}+\left( \frac{1}{2N}+\varepsilon\right)\left| \sin\left( 2N\pi x_{1} \right) \right|+\frac{2}{\left| J_{2} \right|}\sum_{j\in J_{2}} h(y_{j}) \end{aligned} \right.$$

where

$y_{j}=x_{j}-\sin\left( 6\pi x_{1}+\frac{j\pi}{n} \right), j=2,\cdots,n,$

and

$$h\left( t \right)=2t^{2}-\cos\left( 4\pi t \right)+1$$

$J_{1}=\{j|j is odd and 2\leq j\leq n\}$ and $J_{2}=\{j|j is even and 2\leq j\leq n\}$, $N=10,\varepsilon=0.1$and the number of decision variables is $D=30$, and the number of objectives is $M = 2$. The search space is $\left[ 0,1 \right]\times\left[ -1,1 \right]^{n-1}$.

**UF6**

$$min\left\{ \begin{aligned} &f_{1}=x_{1}+max\left\{ 0,2\left( \frac{1}{2N}+\varepsilon\right)\sin\left( 2N\pi x_{1} \right) \right\}+\frac{2}{\left| J_{1} \right|}\left( 4\sum_{j\in J_{1}} y_{j}^{2}-2\prod_{j\in J_{1}} \cos\left( \frac{20y_{j}\pi}{\sqrt{j}} \right)+2 \right) \\ &f_{2}=1-x_{1}+max\left\{ 0,2\left( \frac{1}{2N}+\varepsilon\right)\sin\left( 2N\pi x_{1} \right) \right\}+\frac{2}{\left| J_{2} \right|}\left( 4\sum_{j\in J_{1}} y_{j}^{2}-2\prod_{j\in J_{2}} \cos\left( \frac{20y_{j}\pi}{\sqrt{j}} \right)+2 \right) \end{aligned} \right.$$

where

$y_{j}=x_{j}-\sin\left( 6\pi x_{1}+\frac{j\pi}{n} \right), j=2,\cdots,n,$

$J_{1}=\{j|j is odd and 2\leq j\leq n\}$ and $J_{2}=\{j|j is even and 2\leq j\leq n\}$, $N=2,\varepsilon=0.1$and the number of decision variables is $D=30$, and the number of objectives is $M = 2$. The search space is $\left[ 0,1 \right]\times\left[ -1,1 \right]^{n-1}$.

**UF7**

$$min\left\{ \begin{aligned} &f_{1}=\sqrt[5]{x_{1}}+\frac{2}{\left| J_{1} \right|}\sum_{j\in J_{1}} h(y_{j}) \\ &f_{2}=1-\sqrt[5]{x_{1}}+\frac{2}{\left| J_{2} \right|}\sum_{j\in J_{2}} h(y_{j}) \end{aligned} \right.$$

where

$$y_{j}=x_{j}-\sin\left( 6\pi x_{1}+\frac{j\pi}{n} \right), j=2,\cdots,n,$$

$J_{1}=\{j|j is odd and 2\leq j\leq n\}$ and $J_{2}=\{j|j is even and 2\leq j\leq n\}$, and the number of decision variables is $D=30$, and the number of objectives is $M = 2$. The search space is $\left[ 0,1 \right]\times\left[ -1,1 \right]^{n-1}$.

**UF8**

$$min\left\{ \begin{aligned} f_{1}=\cos(0.5x_{1}\pi)\cos(0.5x_{2}\pi)+\frac{2}{\left| J_{1} \right|}\sum_{j\in J_{1}} \left[ x_{j}-2x_{2}sin (2\pi x_{1}+\frac{j\pi}{n}) \right]^{2} \\ f_{2}=\cos(0.5x_{1}\pi)\cos(0.5x_{2}\pi)+\frac{2}{\left| J_{2} \right|}\sum_{j\in J_{2}} \left[ x_{j}-2x_{2}sin (2\pi x_{1}+\frac{j\pi}{n}) \right]^{2} \\ &f_{3} =\sin0.5x_{1}\pi+\frac{2}{\left| J_{3} \right|}\sum_{j\in J_{3}} \left[ x_{j}-2x_{2}sin (2\pi x_{1}+\frac{j\pi}{n}) \right]^{2} \end{aligned} \right.$$

where

$J_{1}=\left\{ j \right|3\leq j\leq n,and j-1 is a multiplication of 3\}$*,*

$J_{2}=\left\{ j \right|3\leq j\leq n,and j-2 is a multiplication of 3\}$,

$J_{3}=\left\{ j \right|3\leq j\leq n,and j is a multiplication of 3\}$,

the number of decision variables is $D=30$, and the number of objectives is $M = 3$. The search space is $\left[ 0,1 \right]^{2}\times\left[ -2,2 \right]^{n-2}$.

**UF9**

$$min\left\{ \begin{aligned} f_{1} =0.5\left[ max\left\{ 0,\left( 1+\varepsilon\right)\left( 1-4\left( 2x_{1}-1 \right)^{2} \right) \right\}+2x_{1} \right]x_{2}+\frac{2}{\left| J_{1} \right|}\sum_{j\in J_{1}} \left[ x_{j}-2x_{2}sin (2\pi x_{1}+\frac{j\pi}{n}) \right]^{2} \\ f_{2}=0.5\left[ max\left\{ 0,\left( 1+\varepsilon\right)\left( 1-4\left( 2x_{1}-1 \right)^{2} \right) \right\}-2x_{1}+2 \right]x_{2}+\frac{2}{\left| J_{2} \right|}\sum_{j\in J_{2}} \left[ x_{j}-2x_{2}sin (2\pi x_{1}+\frac{j\pi}{n}) \right]^{2} \\ &f_{3} =1-x_{2}+\frac{2}{\left| J_{3} \right|}\sum_{j\in J_{3}} \left[ x_{j}-2x_{2}sin (2\pi x_{1}+\frac{j\pi}{n}) \right]^{2} \end{aligned} \right.$$

where

$J_{1}=\left\{ j \right|3\leq j\leq n,and j-1 is a multiplication of 3\}$*,*

$J_{2}=\left\{ j \right|3\leq j\leq n,and j-2 is a multiplication of 3\}$,

$J_{3}=\left\{ j \right|3\leq j\leq n,and j is a multiplication of 3\}$,

$\varepsilon=0.1$, $\varepsilon$ can take any other positive values. The number of decision variables is $D=30$, and the number of objectives is $M = 3$. The search space is $\left[ 0,1 \right]^{2}\times\left[ -2,2 \right]^{n-2}$.
